# *N*,*N*-Dimethyl-anthranilic Acid from *Calvatia nipponica* Mushroom Fruiting Bodies Induces Apoptotic Effects on MDA-MB-231 Human Breast Cancer Cells

**DOI:** 10.3390/nu15143091

**Published:** 2023-07-10

**Authors:** Dahae Lee, Seulah Lee, Yoon Seo Jang, Rhim Ryoo, Jung Kyu Kim, Ki Sung Kang, Ki Hyun Kim

**Affiliations:** 1 College of Korean Medicine, Gachon University, Seongnam 13120, Republic of Korea; 2 School of Pharmacy, Sungkyunkwan University, Suwon 16419, Republic of Korea; 3 Department of Oriental Medicine Biotechnology, College of Life Sciences, Kyung Hee University, Yongin 17104, Republic of Korea; 4 Special Forest Products Division, Forest Bioresources Department, National Institute of Forest Science, Suwon 16631, Republic of Korea; 5 School of Chemical Engineering, Sungkyunkwan University, Suwon 16419, Republic of Korea

**Keywords:** *Calvatia nipponica*, *N*,*N*-dimethyl-anthranilic acid, breast cancer cells, MDA-MB-231, apoptosis

## Abstract

Breast cancer ranks among the most prevalent malignancies affecting women worldwide, and apoptosis-targeting drugs are attractive candidates for the treatment of cancer. In the current study, we investigated the in vitro cytotoxicity of the mushroom *Calvatia nipponica* in human breast cancer cells (MDA-MB-231), identified potential antitumor compounds through bioactivity-guided isolation, and elucidated the antitumor, pro-apoptotic molecular mechanisms of the identified bioactive compounds. *C. nipponica* is edible when young, and it has been used as a food source as well as a traditional medicine in wound dressings. However, only a limited number of studies have reported its chemical composition and biological activities. In the screening test, the methanol extract of *C. nipponica* fruiting bodies exhibited cytotoxicity against MDA-MB-231 cells. Bioactivity-guided fractionation of the methanol (MeOH) extract and chemical investigation of the active fractions resulted in the isolation of fourteen compounds (**1**–**14**), including six alkaloids (**1**–**3**, **5**, **7**, and **8**), two phenolic compounds (**4** and **6**), one fatty acid (**9**), and five steroids (**10**–**14**). The structures of the isolated compounds were determined using NMR spectroscopic methods, liquid chromatography–mass spectrometry, and comparison of data with previously reported values. The isolated compounds (**1**–**14**) were tested for cytotoxicity against MDA-MB-231 cells, where compound **1**, i.e., *N*,*N*-dimethyl-anthranilic acid, exhibited the most significant cytotoxicity against MDA-MB-231 cells, with an IC_50_ value of 90.28 ± 4.23 μM and apoptotic cell death of 56.01% ± 2.64% at 100 μM. Treatment with compound **1** resulted in an upregulation of protein levels, including cleaved caspase-8, cleaved poly (ADP-ribose) polymerase, Bcl-2-associated X protein (Bax), cleaved caspase-3, cleaved caspase-9, Bad, and Cytochrome *c*, but decreased the levels of B-cell lymphoma 2 (Bcl-2). Overall, these results indicate that *N*,*N*-dimethyl-anthranilic acid (**1**) may have anti-breast cancer activity and is probably involved in the induction of apoptosis mediated by extrinsic and intrinsic signaling pathways.

## 1. Introduction

Breast cancer is the most commonly diagnosed cancer in women around the world [1]. However, the existing drugs for targeted breast cancer therapy are known to have multiple adverse effects [2]. Cisplatin, a representative chemotherapy drug that is commonly used to treat various types of cancer, is well known for its nephrotoxicity, and toxicity in other organs such as the liver, lungs, and nervous system are reported [3]. Thus, research efforts continue to develop drugs that maximize anticancer effects on breast cancer cells without causing side effects in normal cells. The normal growth of breast tissue is controlled by the balance between apoptosis-mediated cell death and cell proliferation, and breast cancer growth results from uncontrolled proliferation while resisting cell death [4]. The modulation of apoptosis signaling pathways has been demonstrated to comprise key events in the activity of anticancer drugs [5]. Natural products are rich sources of anticancer drugs, where more than half of the currently available drugs for cancer therapy are compounds isolated from or related to natural products [6].

Mushrooms are excellent natural sources of bioactive compounds, particularly in the development of novel anticancer agents such as lentinan, grifolin, and *Ganoderma lucidum* polysaccharides [7]. Several studies have identified bioactive components of the *Calvatia* genus. According to previous studies, peptides and a protein, namely calcaelin, isolated from *Calvatia caelata*, have been reported to exhibit antiproliferative effects in human breast cancer cells (MDA-MB-231) [8,9], and calvacin from *Calvatia gigantea* is reported to have antitumor effects in experimental animals [10]. *Calvatia nipponica* (Agaricaceae) is edible when young, and it has been used as a food source as well as a traditional medicine in wound dressings [11,12,13]. This mushroom is an extremely rare species of the *Calvatia* genus. Thus, its chemical constituents as well as biological activities have not yet been fully investigated. Our previous studies indicated several bioactivities of compounds from *C. nipponica*, where some of its alkaloids showed inhibition on angiogenesis in human umbilical vein endothelial cells (HUVECs) [11], and a fatty acid methyl ester exhibited inhibitory effects on nitric oxide (NO) production in lipopolysaccharide (LPS)-stimulated macrophages (RAW264.7) [12]. Furthermore, ergosterol peroxide and cyathisterol from *C. nipponica* were found to exhibit antiestrogenic activity in human breast cancer cells (MCF-7) [13].

In continuation of searching for interesting bioactive components with novel structures from diverse natural products [14,15,16,17,18], we examined antitumor compounds from the relatively unexplored rare mushroom, *C. nipponica*. In a screening test, the methanol (MeOH) extract of the fruiting bodies of *C. nipponica* exhibited cytotoxicity toward human breast cancer cells (MDA-MB-231). Subsequent bioactivity-guided isolation of the MeOH extract led to the isolation of fourteen compounds (**1**–**14**), including six alkaloids (**1**–**3**, **5**, **7**, and **8**), two phenolics (**4** and **6**), one fatty acid (**9**), and five steroids (**10**–**14**). The isolated compounds were tested for cytotoxicity against MDA-MB-231 cells to identify the antitumor compounds. Herein, we describe the isolation and structural elucidation of compounds **1**–**14**, along with their antitumor potentials and the underlying molecular mechanisms of the identified antitumor compounds.

## 2. Materials and Methods

### 2.1. General Experimental Procedures

The following instrumental techniques were employed for spectroscopic and chromatographic analysis:

Infrared (IR) spectra: Recorded using an IFS-66/s FT-IR spectrometer (Bruker, Karlsruhe, Germany). Ultraviolet (UV) spectra: Acquired using an Agilent 8453 UV–visible spectrophotometer (Agilent Technologies, Santa Clara, CA, USA). Circular dichroism (CD) spectra: Recorded using a JASCO J-810 spectropolarimeter (Jasco, Tokyo, Japan). High-resolution (HR) electrospray ionization (ESI) mass spectra: Recorded on a UPLC-QTOF Xevo G2-S mass spectrometer (Waters Corp., Milford, CT, USA). NMR spectra: Measured using a Bruker AVANCE III 700 NMR spectrometer operating at 700 MHz (^1^H) and 175 MHz (^13^C) (Bruker, Billerica, MA, USA). Preparative high-performance liquid chromatography (HPLC): Conducted using a Waters 1525 binary HPLC pump with a Waters 996 photodiode array detector (Waters Corp., Milford, MA, USA) equipped with an Agilent Eclipse C18 column (21.2 × 250 mm i.d., flow rate: 5 mL/min). Semi-preparative HPLC: Conducted using a Shimadzu Prominence HPLC System with SPD-20A/20AV series Prominence HPLC UV–vis detectors (Shimadzu, Tokyo, Japan) equipped with a Phenomenex Luna phenyl-hexyl column (250 × 10 mm i.d., flow rate: 2 mL/min). Liquid chromatography–mass spectrometry (LC-MS) analysis: Performed on an Agilent 1200 series HPLC system with a diode array detector and a 6130 Series ESI mass spectrometer using an analytical Kinetex C18 100 Å column (100 mm × 2.1 mm i.d., 5 μm) (Phenomenex, Torrance, CA, USA). Column chromatography: Performed using silica gel 60, 230–400 mesh; reverse-phase (RP)-C_18_ silica gel, 230–400 mesh (Merck, Darmstadt, Germany); silica Sep-Pak Vac (6 cc) cartridges; and C18 Sep-Pak Vac (6 cc) cartridges (Waters, Milford, MA, USA). Thin-layer chromatography (TLC): Conducted using precoated silica gel F_254_ plates and RP-18 F_254s_ plates (Merck, Rahway, NJ, USA). TLC spots were detected under UV light after dipping in anisaldehyde–sulfuric acid and heating.

### 2.2. Fungus Material

Fresh *C. nipponica* fruiting bodies were collected in August 2014 at Jeonju, Jeollabuk-do, Republic of Korea. One of the authors (K.H. Kim) authenticated a voucher specimen (HCCN26287) of the mushroom, which was deposited at the Herbarium Conservation Center of the National Institute of Agricultural Sciences, RDA, Republic of Korea.

### 2.3. Extraction and Separation of the Compounds

The air-dried fruiting bodies of *C. nipponica* (200 g) were subjected to extraction using 80% aqueous MeOH. The extraction was performed three times, each lasting for 2.0 L of solvent over a span of three days at room temperature. The resulting extracts were filtered, and the filtrate was then evaporated under reduced pressure using a rotavapor. This process yielded a MeOH extract weighing 13.8 g.

The cytotoxicity of the MeOH extract against human breast cancer cells (MDA-MB-231) was evaluated, revealing significant cytotoxic effects. To conduct bioactivity-guided fractionation, the MeOH extract was partitioned with different solvents: hexane, dichloromethane (CH_2_Cl_2_), ethyl acetate (EtOAc), and *n*-butanol (*n*-BuOH). This process yielded a hexane-soluble layer (HL) weighing 1.1 g, a CH_2_Cl_2_-soluble layer weighing 0.4 g, an EtOAc-soluble layer weighing 0.8 g, and an *n*-BuOH-soluble layer (BL) weighing 3.1 g. Due to similar patterns observed in TLC analysis, the CH_2_Cl_2_-soluble and EtOAc-soluble layers were combined to obtain a consolidated CH_2_Cl_2_- and EtOAc-soluble layer (CEL). The cytotoxicity of HL, BL, and CEL fractions against the MDA-MB-231 cell line was evaluated. CEL fraction exhibited significant suppression of cell proliferation with an IC_50_ value of 77.25 ± 2.05 μg/mL. At a concentration of 100 μg/mL, the cell viability was reduced to 72.51% ± 2.41% and 52.11% ± 3.21% by treatment with the BL and HL fractions, respectively. Based on these findings, the active fractions HL and CEL were further investigated to identify the chemical constituents responsible for their antiproliferative activity against the MDA-MB-231 cell line.

Fraction HL (1.1 g) underwent fractionation through silica gel column chromatography, utilizing a gradient solvent system of hexane/EtOAc (20:1 to 1:1). This process resulted in the isolation of 12 fractions (H1–H12). Fractions H1 (46.8 mg) and H2 (7.6 mg) were combined based on their TLC patterns and subjected to purification using semi-preparative HPLC with a 93% MeOH isocratic solvent system. This led to the isolation of compound **9** (9.7 mg, *t*_R_ 36.0 min). Fraction H5 (13.4 mg) was also purified using semi-preparative HPLC (98% MeOH), yielding compound **13** (0.9 mg, *t*_R_ 29.5 min). Additionally, fraction H8 (120.5 mg) was purified through semi-preparative HPLC and eluted with 85% acetonitrile, resulting in the isolation of compounds **10** (1.4 mg, *t*_R_ 63.5 min), **11** (2.5 mg, *t*_R_ 68.5 min), and **12** (4.3 mg, *t*_R_ 71.0 min). Compound **14** (0.4 mg, *t*_R_ 24.5 min) was purified from fraction H9 (39.5 mg) using semi-preparative HPLC with 85% acetonitrile.

Fraction CEL (1.2 g) was subjected to silica gel column chromatography using a gradient solvent system of CH_2_Cl_2_/MeOH (30:1 to 0:1). This process resulted in the separation of eight fractions (C1–C8). Fraction C3 (170.8 mg) from the chromatography was first eluted with 100% MeOH on a C_18_ Sep-Pak column. Subsequently, it underwent purification using preparative HPLC with a 50% MeOH solvent system, leading to the isolation of compound **5** (2.1 mg, *t*_R_ 29.0 min). Fraction C4 (295.9 mg) was also fractionated using preparative HPLC, utilizing a 65% MeOH solvent system. This step yielded eight fractions (C41–C48). Further purification of fraction C41 (164.3 mg) was carried out using semi-preparative HPLC with a 17% MeOH solvent system, resulting in the isolation of compounds **1** (87.3 mg, *t*_R_ 13.5 min), **2** (0.7 mg, *t*_R_ 16.0 min), **3** (0.7 mg, *t*_R_ 19.0 min), and **4** (1.7 mg, *t*_R_ 28.3 min). In addition, fraction C5 (30.0 mg) underwent purification using semi-preparative HPLC with a 20% MeOH solvent system, leading to the isolation of compound **6** (0.4 mg, *t*_R_ 11.2 min). Purification of fraction C6 (33.2 mg) using semi-preparative HPLC with a 15% MeOH solvent system resulted in the isolation of compounds **7** (0.7 mg, *t*_R_ 13.5 min) and **8** (10.7 mg, *t*_R_ 25.0 min).

### 2.4. Cell Culture

Human breast cancer cells (MDA-MB-231), mouse skeletal muscle cells (C2C12), human lung cancer cells (A549), and human hepatocellular carcinoma cells (HepG2) were obtained from the American Type Culture Collection (Rockville, MD, USA). MDA-MB-231 and A549 cells were cultured in Roswell Park Memorial Institute (RPMI) 1640 medium (Cellgro, Manassas, VA, USA), while C2C12 and HepG2 cells were cultured in Dulbecco’s modified Eagle’s medium (DMEM, Cellgro, Lincoln, NE, USA). All culture media were supplemented with 1% penicillin/streptomycin (Gibco BRL, Carlsbad, MD, USA) and 10% fetal bovine serum (Gibco BRL, Carlsbad, MD, USA). The cells were maintained in a 37 °C incubator with a humidified atmosphere containing 5% CO_2_ and 95% air.

### 2.5. Cell Viability Assay

MDA-MB-231, C2C12, A549, and HepG2 cells were diluted to a concentration of 1 × 10^4^ cells/well and seeded in 96-well plates. After incubation for 24 h, the cells were treated with samples at concentrations of 12.5, 25, 50, and 100 μM for 24 h. Dimethyl sulfoxide (DMSO) diluted to a concentration of 0.5% in RPMI 1640 medium was used as the vehicle control. To measure cell viability, Ez-Cytox reagent (Daeil Lab Service Co., Seoul, Republic of Korea) was added to each well. After incubation for 1 h, the absorbance at 490 nm was recorded using a PowerWave XS microplate reader (Bio-Tek Instruments, Winooski, VT, USA). The Ez-Cytox assay measures cell mitochondrial activity based on the conversion of water-soluble tetrazolium 1 to insoluble formazan.

### 2.6. Annexin V Staining

MDA-MB-231 cells were diluted to a concentration of 4 × 105 cells/well in RPMI 1640 medium and seeded in 6-well plates. After incubation for 24 h, the cells were treated with compound 1 at concentrations of 50 and 100 μM for 24 h. Dimethyl sulfoxide (DMSO) diluted to a concentration of 0.5% in RPMI 1640 medium was used as the vehicle control. The cells were collected and washed with binding buffer (Life Technologies, Carlsbad, CA, USA). Subsequently, the cells were stained in binding buffer with annexin V Alexa Fluor 488 (Invitrogen, Temecula, CA, USA) for 30 min in the dark. Annexin-V-positive apoptotic cells were quantified using a Tali Image-Based Cytometer (Invitrogen, Temecula, CA, USA).

### 2.7. Western Blotting Analysis

Equal amounts of protein (20 μg) were loaded onto 10% sodium dodecyl sulfate–polyacrylamide gel electrophoresis (SDS-PAGE) gels and separated. The proteins were then transferred onto polyvinylidene difluoride (PVDF) membranes obtained from Pall Corporation (Washington, NY, USA). Primary antibodies against specific proteins were purchased from Cell Signaling Technology (Danvers, MA, USA). The membranes were incubated overnight on ice with the primary antibodies at the following dilutions: cleaved caspase-8 (1:1000), Bcl-2-associated X protein (Bax, 1:1000), cleaved caspase-9 (1:1000), cleaved caspase-3 (1:1000), poly (ADP-ribose) polymerase (PARP, 1:1000), Bad (1:1000), Cytochrome *c* (1:1000), and glyceraldehyde-3-phosphate dehydrogenase (GAPDH, 1:1000). After washing, the membranes were probed with the appropriate secondary antibody (1:2000) for approximately 60 min on ice. Visualization of the membranes was performed using a chemiluminescence system of FUSION Solo (PEQLAB Biotechnologie GmbH, Erlangen, Germany). Band intensities were measured with Gel Analyzer in the open-source image analysis software FIJI version 2.1.1.

### 2.8. Statistical Analysis

The experiment was repeated independently three times on different days. Statistical analysis was conducted using one-way analysis of variance (ANOVA), followed by multiple comparisons with Bonferroni corrections. A significance level of *p* < 0.05 was considered statistically significant. All data analyses were performed using SPSS Statistics ver. 19.0 (SPSS Inc., Armonk, NY, USA).

## 3. Results

### 3.1. Bioactivity-Guided Fractionation

**Figure 1:**
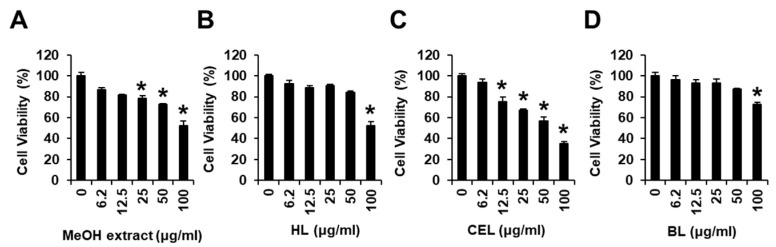
Effects of the methanol (MeOH) extract of *Calvatia nipponica* fruiting bodies and hexane (HL), consolidated dichloromethane and ethyl acetate (CEL), and *n*-butanol (BL) phase-partitioning fractions on human breast cancer (MDA-MB-231) cell viability. MDA-MB-231 cells were treated with (**A**) MeOH extract, (**B**) HL, (**C**) CEL, or (**D**) BL (6.2, 12.5, 25, 50, and 100 μg/mL) for 24 h. MDA-MB-231 cells were treated with culture media containing 0.5% dimethyl sulfoxide as the vehicle control. The Ez-Cytox cell viability assay was used to assess cell viability. Data are presented as the mean ± standard error of the mean (SEM). n = 3; * *p* < 0.05 compared with the control (0 µg/mL).

In our screening of the MeOH extract of *C. nipponica* for in vitro anticancer activity, cell viability assays were conducted against MDA-MB-231 human breast cancer cells. Compared to control, the cell viability was suppressed to 78.79% ± 2.36%, 72.96% ± 0.26%, and 52.11% ± 4.51% by treatment with the MeOH extract at 25, 50, and 100 μg/mL, respectively (Figure 1A). The MeOH extract was sequentially solvent-partitioned using hexane, CH_2_Cl_2_, EtOAc, and *n*-BuOH. This yielded the solvent phases HL, BL, and CEL based on their TLC analysis. These fractions were evaluated for their cytotoxicity in MDA-MB-231 cells to determine the antitumor compounds that contribute to cytotoxicity of the MeOH extract (Figure 1). Assessment of viable cells using the Ez-Cytox cell viability assay revealed that the CEL fraction significantly suppressed cell proliferation in a dose-dependent manner (Figure 1, IC_50_: 77.25 ± 2.05 μg/mL). The other fractions showed no efficacy in inhibiting MDA-MB-231 cell viability, although it was suppressed to 72.51% ± 2.41% and 52.11% ± 3.21%, respectively, by treatment with BL and HL fractions at 100 μg/mL compared to control (Figure 1). Based on these results, we explored the most active fraction, namely CEL, and another active fraction, namely HL, to identify cytotoxic compounds that possibly contribute to cytotoxicity against MDA-MB-231 cells.

### 3.2. Isolation and Structural Elucidation of Compounds ***1**–**14***

**Figure 2:**
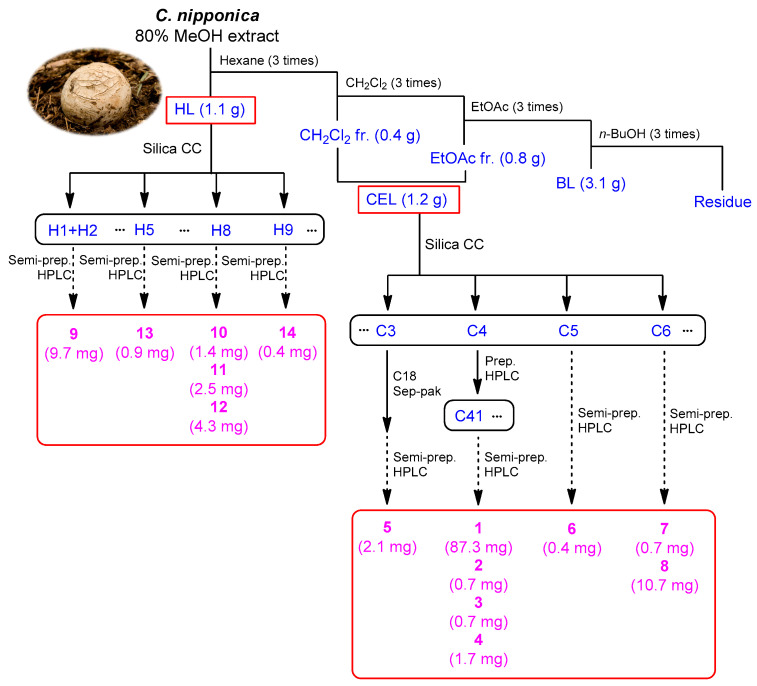
Bioactivity-guided isolation of compounds **1**–**14**. CC, column chromatography; fr., fraction; HL, hexane layer; BL, *n*-butanol layer; CEL, consolidated dichloromethane and ethyl acetate layer.

Based on bioactivity-guided fractionation, the active fractions CEL and HL were investigated to identify the constituents that mediate in vitro cytotoxicity against MDA-MB-231 cells. The LC-MS analysis combined with in-house UV library reference was utilized to investigate potential bioactive compounds. The active fractions were subjected to extensive chemical analysis using repeated column chromatography and HPLC, which led to the isolation of 14 compounds, including six alkaloids (**1**–**3**, **5**, **7**, and **8**), two phenolics (**4** and **6**), one fatty acid (**9**), and five steroids (**10**–**14**) (Figure 2).

The comparison of NMR spectroscopic data (Figures S1, S2, S4–S7, S9, S10, S12, S13, S15, S17, S19, S20, S22, S24–S26 and S28–S31) with those previously reported, along with LC-MS data (Figures S3, S8, S11, S14, S16, S18, S21, S23 and S27), allowed the identification of the isolated compounds as *N*,*N*-dimethyl-anthranilic acid (**1**) [12], *N*-(4-hydroxyphenyl)acetamide (**2**) [19], acetaminophen (**3**) [20], 5,7-dihydroxyisobenzofuran-1(3*H*)-one (**4**) [12], *N*-methylanthranilic acid (**5**) [21], 3,5-dihydroxybenzyl alcohol (**6**) [22], nicotinamide (**7**) [23], 2,3-dihydro-4,6-dihydroxy-1*H*-isoindol-1-one (**8**) [24], (7*Z*,10*Z*)-7,10-octadecadienoic acid methyl ester (**9**) [25], 9,11-dehydroergosterol peroxide (**10**) [26], calvatianone (**11**) [13], ergosterol peroxide (**12**) [26], cyathisterol (**13**) [27], and dankasterone B (**14**) [28] (Figure 3).

### 3.3. Effects of Isolated Compounds s ***1**–**14*** on Viability of MDA-MB-231 Cells

**Figure 4:**
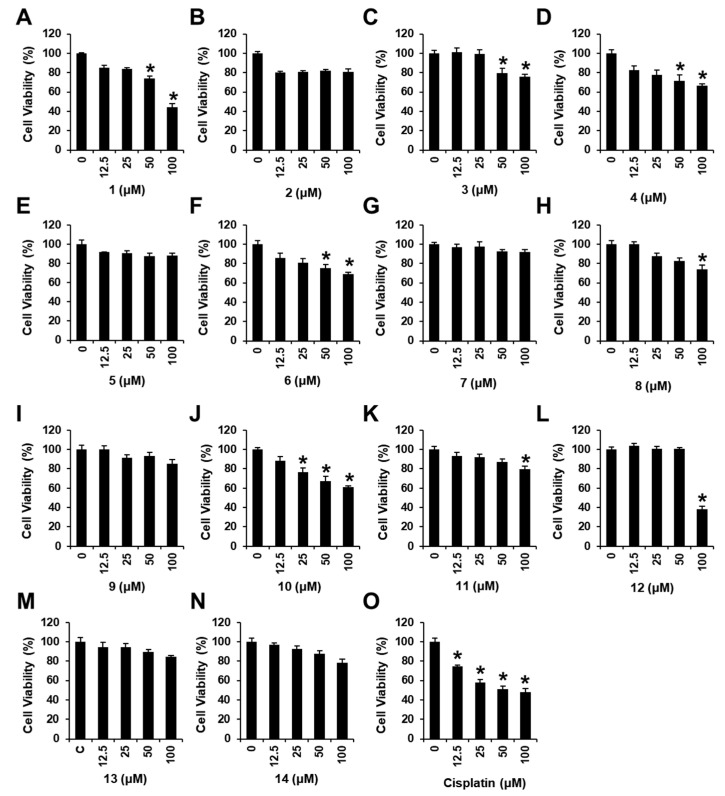
Effects of compounds **1**–**14** on human breast cancer (MDA-MB-231) cell viability. MDA-MB-231 cells were treated with compounds **1** (**A**), **2** (**B**), **3** (**C**), **4** (**D**), **5** (**E**), **6** (**F**), **7** (**G**), **8** (**H**), **9** (**I**), **10** (**J**), **11** (**K**), **12** (**L**), **13** (**M**), **14** (**N**), and cisplatin (**O**) (12.5, 25, 50, and 100 μM) for 24 h. MDA-MB-231 cells were treated with 0.5% dimethyl sulfoxide in cell culture media as vehicle controls. The MDA-MB-231 cell viability was assessed using the Ez-Cytox cell viability assay. Data are presented as the mean ± standard error of the mean (SEM). n = 3; * *p* < 0.05 compared with the control (0 µM).

**Figure 5:**
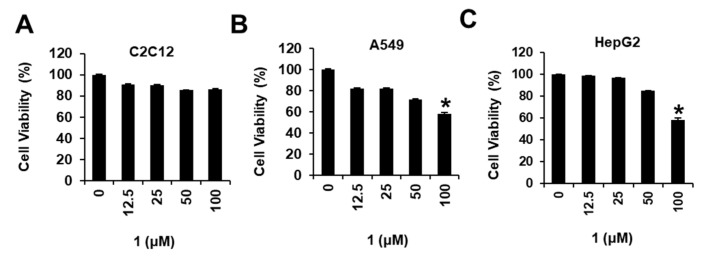
Effects of compound **1** on cell viability in mouse skeletal muscle cells (C2C12), human lung cancer cells (A549), and human hepatocellular carcinoma cells (HepG2). (**A**) Mouse skeletal muscle cells (C2C12), (**B**) human lung cancer cells (A549), and (**C**) human hepatocellular carcinoma cells (HepG2) were treated with compound **1** (12.5, 25, 50, and 100 μM) for 24 h. The cells were treated with 0.5% dimethyl sulfoxide in cell culture media as vehicle controls. The cell viability was assessed using the Ez-Cytox cell viability assay. Data are presented as the mean ± standard error of the mean (SEM). n = 3; * *p* < 0.05 compared with the control (0 µM).

We examined the effects of compounds **1**–**14** on the viability of MDA-MB-231 cells (Figure 4) using an Ez-Cytox cell viability assay. Several compounds tested showed cytotoxic effects against MDA-MB-231 cells, decreasing cell viability (44.12 ± 0.02% for **1**, 76.02 ± 0.01% for **3**, 66.57 ± 1.94% for **4**, 69.03 ± 0.01% for **6**, 74.39 ± 0.02% for **8**, 61.39 ± 0.01% for **10**, 79.70 ± 0.01% for **11**, and 38.08 ± 0.01% for **12**) at 100 μM. Among the isolated compounds, *N*,*N*-dimethyl-anthranilic acid (**1**) exhibited the most significant cytotoxicity against MDA-MB-231 cells. The IC_50_ value of **1** was 90.28 ± 4.23 μM, while that of cisplatin (positive control) was 99.13 ± 2.91 μM (Figure 4). In addition, we examined the effects of *N*,*N*-dimethyl-anthranilic acid (**1**) on the viability in the other cell lines, including mouse skeletal muscle cells (C2C12), human lung cancer cells (A549), and human hepato-cellular carcinoma cells (HepG2). As a result, *N*,*N*-dimethyl-anthranilic acid (**1**) did not affect the viability of C2C12, A549, and HepG2 (IC_50_ > 100 μM) (Figure 5). *N*,*N*-dimethyl-anthranilic acid (**1**) was also tested for cytotoxicity against other breast cancer cell lines (Bt549, HCC70, and MDA-MB-468); however, no significant cytotoxicity was observed (IC_50_ > 100 μM). Therefore, *N*,*N*-dimethyl-anthranilic acid (**1**) was selected for subsequent experiments on MDA-MB-231 human breast cancer cells.

### 3.4. Image-Based Cytometric Analysis of N,N-Dimethyl-Anthranilic Acid (***1***)

**Figure 6:**
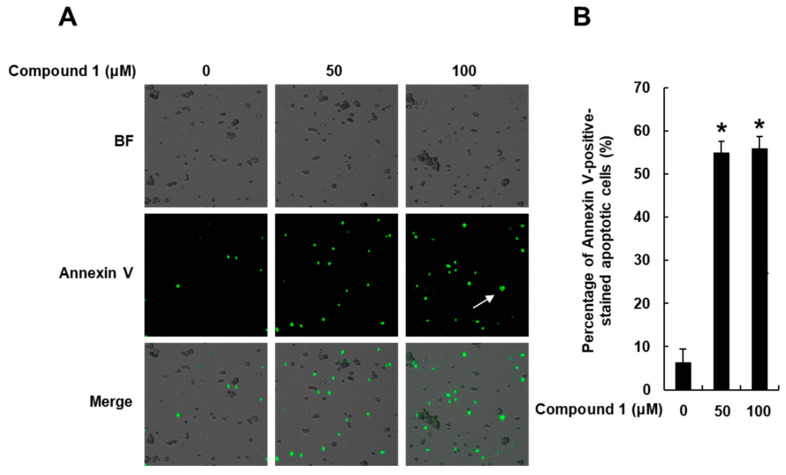
Effects of compound **1** on apoptotic death of human breast cancer (MDA-MB-231) cells. MDA-MB-231 cells were treated with **1** (50 and 100 μM) for 24 h. The cells were treated with 0.5% dimethyl sulfoxide in cell culture media as vehicle controls: (**A**) visualization of apoptotic cells stained with annexin V (40× magnification). Apoptotic cells stained with annexin V are marked by a white arrow. (**B**) Bar graph representing percentage of apoptotic cells. Data are presented as the mean ± standard error of the mean (SEM). n = 3; * *p* < 0.05 compared with the control (0 µM). BF, bright field.

Next, image-based cytometric analysis was performed to evaluate whether treatment with *N*,*N*-dimethyl-anthranilic acid (**1**) increased the number of annexin-V-positive apoptotic cells. After treatment with compound **1** (50 and 100 μM), the MDA-MB-231 cells were stained with annexin V Alexa Fluor 488. The results showed that compound **1** leads to apoptotic cell death. As shown in Figure 6, the percentage of annexin-V-positive cells defining apoptotic cell death was significantly increased to 55.03% ± 2.64% and 56.01% ± 2.64% by treatment with 50 and 100 μM of compound **1**, respectively. Apoptosis in the control cells was 6.33% ± 3.05%.

### 3.5. Effects of N,N-Dimethyl-Anthranilic Acid (***1***) on Apoptosis Signaling Pathways in MDA-MB-231 Cells

**Figure 7:**
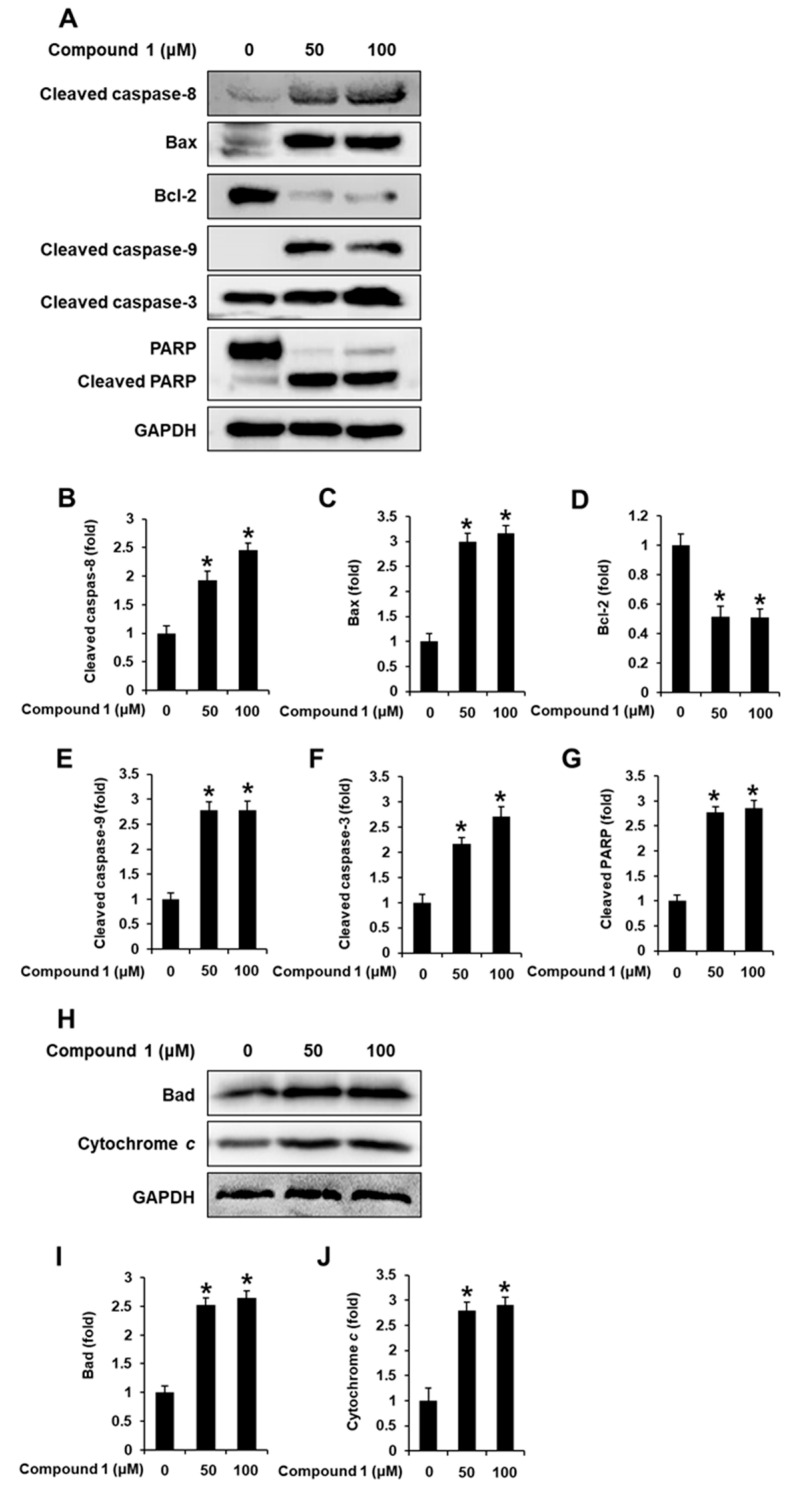
Effects of compound **1** on apoptosis signaling pathways in human breast cancer (MDA-MB-231) cells. MDA-MB-231 cells were treated with compound **1** (50 and 100 μM) for 24 h. MDA-MB-231 cells were treated with 0.5% dimethyl sulfoxide in cell culture media as vehicle controls: (**A**) protein levels of cleaved caspase-8, Bax, Bcl-2, cleaved caspase-9, cleaved caspase-3, cleaved PARP, and GAPDH. (**B**–**G**) Each bar graph presents the densitometric quantification of protein levels. (**H**) Protein levels of Bad, Cytochrome *c*, and GAPDH. (**I**,**J**) Each bar graph presents the densitometric quantification of protein levels. Data are presented as the mean ± standard error of the mean (SEM). n = 3; * *p* < 0.05 compared with the control (0 µM).

Potential molecular mechanisms of the pro-apoptotic effect of *N*,*N*-dimethyl-anthranilic acid (**1**) on MDA-MB-231 cells were evaluated. Treatment with **1** (50 and 100 μM) increased the protein levels of cleaved caspase-9, cleaved caspase-8, cleaved caspase-3, Bax, cleaved PARP, Bad, and Cytochrome *c* while decreasing those of Bcl-2 (Figure 7).

## 4. Discussion

Due to the rarity of *C. nipponica*, its chemical components and biological activities have been less studied than those of other medicinal mushrooms. In our study searching for new bioactive natural products that exhibit apoptosis-inducing activities, we investigated the efficiency of the MeOH extract of *C. nipponica* against the MDA-MB-231 human breast cancer cell line. As a result, we observed that the extract suppressed cell viability in a concentration-dependent manner. Chemical analysis was performed to isolate 14 chemical constituents from the hexane-soluble layer (HL) and the combined fraction of the CH_2_Cl_2_- and EtOAc-soluble layers (CEL). Among the isolated compounds, *N*,*N*-dimethyl-anthranilic acid (**1**) exhibited the most significant cytotoxicity against MDA-MB-231 cells.

**Figure 8:**
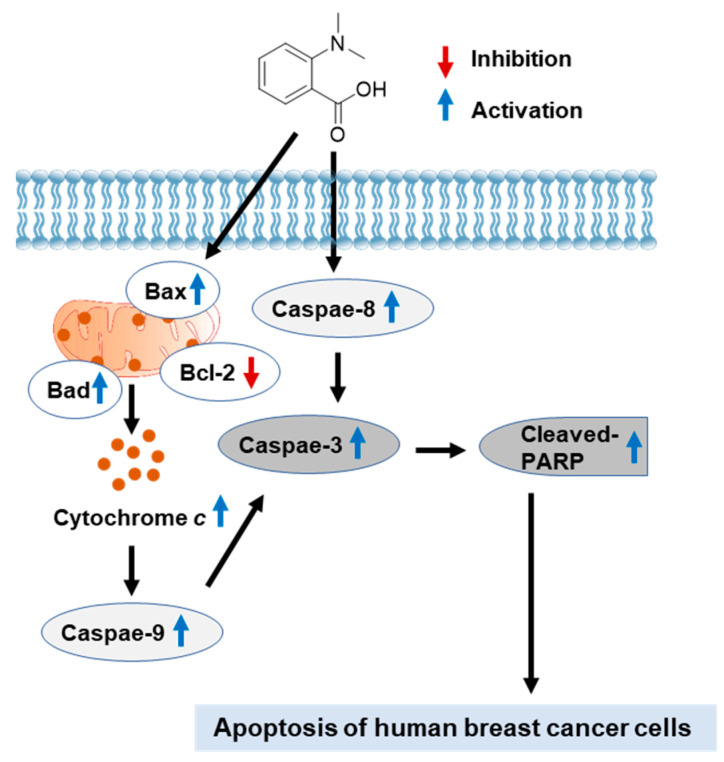
Schematic pathway illustrating the potential role of compound **1** on apoptosis of human breast cancer cells. Bax, Bcl-2-associated X protein; Bcl-2, B-cell lymphoma 2; cleaved PARP, cleaved poly (ADP–ribose) polymerase.

To further confirm the relevance of cytotoxicity of *N*,*N*-dimethyl-anthranilic acid (**1**) to apoptotic cell death, image-based cytometric analysis was performed. The percentage of annexin-V-positive cells representing apoptotic cell death significantly increased following treatment with **1**, confirming that compound **1** induces apoptosis. The recognition that defective apoptosis causing cancer is a critical obstacle to overcome in cancer treatment has led to the discovery of a variety of therapeutic strategies aimed at inducing apoptotic signaling pathways [29,30]. Our data demonstrated that the apoptotic cell death induced by compound **1** was associated with the induction of Bax, Bad, and Cytochrome c, inhibition of Bcl-2, and cleavage of caspase-8, -9, -3, and PARP. These results indicated that treatment with compound **1** induced extrinsic and intrinsic apoptotic signaling pathways (Figure 8). Among the two main signaling pathways of apoptosis, the extrinsic pathway requires caspase 8 recruitment to death domain receptors, which leads to the cleavage of caspase-8 and subsequent cleavage of the downstream executioner caspase-3 [31]. The intrinsic pathway is induced by hypoxia, oxidative stress, and chemotherapeutic agents and leads to mitochondrial dysfunction. The interactions between pro-apoptotic members such as Bax and Bad and anti-apoptotic members such as Bcl-2 control the intrinsic pathway with mitochondrial dysfunction and cytochrome c release [32]. Cytochrome c released from the mitochondria into the cytosol promotes the cleavage of caspase-9 and subsequent cleavage of the downstream executioner caspase-3 [33]. Cleaved caspase-3 is able to cleave PARP, eventually leading to DNA fragmentation and triggering apoptosis [34]. Consequently, the findings of the present study revealed that *N*,*N*-dimethyl-anthranilic acid (**1**) has the potential to treat breast cancers and is predicted to be associated in the induction of apoptosis mediated by extrinsic and intrinsic signaling pathways. The exact mechanism of action and safety assurance of compound **1** requires further investigation in an MDA-MB-231 xenograft mouse model to validate these findings.

In addition to compound **1**, compounds **4**, **6**, and **10** exhibited weak cytotoxicity against human cancer cells. Although compound **4**, i.e., 5,7-dihydroxyisobenzofuran-1(3*H*)-one, lacked cytotoxic activity against tumor cell lines HL-60, MCF-7, A549, SW480, and SMMC-7221, at a concentration of 40 µM, as used in a previous study [35], it exhibited dose-dependent cytotoxicity against MDA-MB-231 cells in the present study. Compound **6**, i.e., 3,5-dihydroxybenzyl alcohol, was reported to have weak cytotoxicity against MCF-7 and A-549 cell lines [36]. Compound **10**, i.e., 9,11-dehydroergosterol peroxide, was reported to have moderate cytotoxicity in MDA-MB-231 cells [37], potent cytotoxicity against MCF-7 cells [38,39], and weak cytotoxicity against HeLa, A-549, and J5 cell lines [40,41]. Furthermore, compound **10** was found to exert cytotoxicity against human lung adenocarcinoma cells, including H1264, H1299, and Calu-6, in addition to A549 [42] and HepG2 [37] cells. The current and previous results indicate that the constituents isolated from *C. nipponica* may be potential sources of new antitumor agents.

## 5. Conclusions

In this study, bioactivity-guided isolation of the MeOH extract of *C. nipponica* fruiting bodies and chemical analysis of the active fractions resulted in the isolation of fourteen compounds (**1**–**14**), including six alkaloids (**1**–**3**, **5**, **7**, and **8**), two phenolics (**4** and **6**), one fatty acid (**9**), and five steroids (**10**–**14**) via LC-MS-based analysis. Among these compounds, compound **1**, i.e., *N*,*N*-dimethyl-anthranilic acid, exhibited the most significant cytotoxic effect against MDA-MB-231 cells. Compound **1** induced apoptotic cell death, as evidenced by increased protein levels of cleaved caspase-8, cleaved caspase-3, Bax, PARP, cleaved caspase-9, Bad, and Cytochrome *c* and decreased protein expression levels of Bcl-2. Although the cytotoxicity of compound **1** against MDA-MB-231 cells is not remarkably potent, the current study provides sufficient evidence that *N*,*N*-dimethyl-anthranilic acid (**1**) has anticancer potential against human breast cancer cells. Furthermore, the current findings can possibly contribute to the exploration of diverse novel, natural products through structural optimization, which can be applied to the treatment of breast cancer.

## Figures and Tables

**Figure 3:**
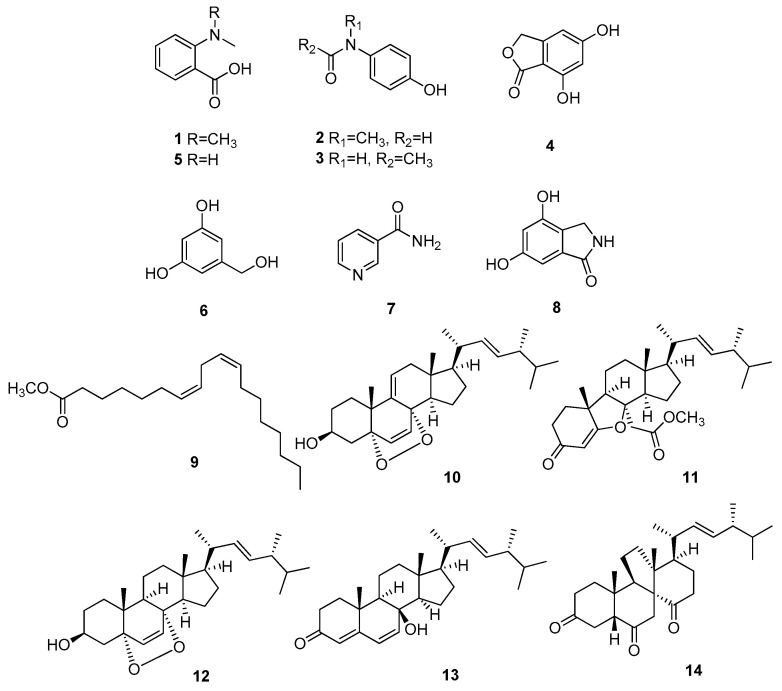
Chemical structures of the compounds (**1**–**14**) isolated from *C. nipponica*.

## Data Availability

Not applicable.

## Supplementary Materials

The following are available online at https://www.mdpi.com/article/10.3390/nu15143091/s1: Figure S1: ^1^H NMR spectrum of **1**; Figure S2: ^13^C NMR spectrum of **1**; Figure S3: ESIMS spectrum of **1**; Figure S4: ^1^H NMR spectrum of **2**; Figure S5: ^13^C NMR spectrum of **2**; Figure S6: ^1^H NMR spectrum of **3**; Figure S7: ^13^C NMR spectrum of **3**; Figure S8: ESIMS spectrum of **3**; Figure S9: ^1^H NMR spectrum of **4**; Figure S10: ^13^C NMR spectrum of **4**; Figure S11: ESIMS spectrum of **4**; Figure S12: ^1^H NMR spectrum of **5**; Figure S13: ^13^C NMR spectrum of **5**; Figure S14: ESIMS spectrum of **5**; Figure S15: ^1^H NMR spectrum of **6**; Figure S16: ESIMS spectrum of **6**; Figure S17: ^1^H NMR spectrum of **7**; Figure S18: ESIMS spectrum of **7**; Figure S19: ^1^H NMR spectrum of **8**; Figure S20: ^13^C NMR spectrum of **8**; Figure S21: ESIMS spectrum of **8**; Figure S22: ^1^H NMR spectrum of **9**; Figure S23: ESIMS spectrum of **9**; Figure S24: ^1^H NMR spectrum of **10**; Figure S25: ^1^H NMR spectrum of **11**; Figure S26: ^13^C NMR spectrum of **11**; Figure S27: ESIMS spectrum of **11**; Figure S28: ^1^H NMR spectrum of **12**; Figure S29: ^1^H NMR spectrum of **13**; Figure S30: ^1^H NMR spectrum of **14**; Figure S31: ^13^C NMR spectrum of **14**.
