# Supplementary material for: N,N-Dimethyl-anthranilic Acid from Calvatia nipponica Mushroom Fruiting Bodies Induces Apoptotic Effects on MDA-MB-231 Human Breast Cancer Cells
Source: Nutrients. 2023 Jul 10;15(14):3091. doi: 10.3390/nu15143091 (PMC10386113; doi:10.3390/nu15143091)
Supplement: Supplementary file 1 [file nutrients-15-03091-s001.zip › nutrients-2486879-supplementary.pdf]

Supplementary Information

N,*N*-Dimethyl-Anthranilic Acid from *Calvatia nipponica* Mushroom Fruiting Bodies Induces Apoptotic Effects on MDA-MB-231 Human Breast Cancer Cells

Dahae Lee [1,†], Seulah Lee [2,3,†], Yoon Seo Jang [2], Rhim Ryoo [4], Jung Kyu Kim [5], Ki Sung Kang [1,*], and Ki Hyun Kim [2,*]

[1] College of Korean Medicine, Gachon University, Seongnam 13120, Korea; pjsldh@naver.com (D.L.)

[2] School of Pharmacy, Sungkyunkwan University, Suwon 16419, Korea; lee.seulah@khu.ac.kr (S.L.); bbj0423@gmail.com (Y.S.J.)

[3] Department of Oriental Medicine Biotechnology, College of Life Sciences, Kyung Hee University, Yongin, 17104, Korea

[4] Special Forest Products Division, Forest Bioresources Department, National Institute of Forest Science, Suwon 16631, Korea; rryoo@korea.kr (R.R.)

[5] School of Chemical Engineering, Sungkyunkwan University, Suwon 16419, Korea; legkim@skku.edu (J.K.K.)

[6] STL company, Yongin 17086, Korea; hkkim@stlcorp.net (H.K.K.)

[*] Correspondence: khkim83@skku.edu (K.H.K.); +82-31-290-7700 (K.H.K.); kkang@gachon.ac.kr (K.S.K.); Tel.: +82-31-750-5402 (K.S.K.)

[†] These authors contributed equally to this study.

Supporting Information Contents:

Figure S1. ^{1}H NMR spectrum of **1** (CD_3OD, 700 MHz)……S4
Figure S2. ^{13}C NMR spectrum of **1** (CD_3OD, 175 MHz)……S5
Figure S3. ESIMS spectrum of **1**……S6
Figure S4. ^{1}H NMR spectrum of **2** (CD_3OD, 700 MHz)……S7
Figure S5. ^{13}C NMR spectrum of **2** (CD_3OD, 175 MHz)……S8
Figure S6. ^{1}H NMR spectrum of **3** (CD_3OD, 700 MHz)……S9
Figure S7. ^{13}C NMR spectrum of **3** (CD_3OD, 175 MHz)……S10
Figure S8. ESIMS spectrum of **3**……S11
Figure S9. ^{1}H NMR spectrum of **4** (CD_3OD, 700 MHz)……S12
Figure S10. ^{13}C NMR spectrum of **4** (CD_3OD, 175 MHz)……S13
Figure S11. ESIMS spectrum of **4**……S14
Figure S12. ^{1}H NMR spectrum of **5** ($CDCl_3$, 700 MHz)……S15
Figure S13. ^{13}C NMR spectrum of **5** ($CDCl_3$, 175 MHz)……S16
Figure S14. ESIMS spectrum of **5**……S17
Figure S15. ^{1}H NMR spectrum of **6** (CD_3OD, 700 MHz)……S18
Figure S16. ESIMS spectrum of **6**……S19
Figure S17. ^{1}H NMR spectrum of **7** ($CDCl_3$, 700 MHz)……S20
Figure S18. ESIMS spectrum of **7**……S21
Figure S19. ^{1}H NMR spectrum of **8** (CD_3OD, 700 MHz)……S22
Figure S20. ^{13}C NMR spectrum of **8** (CD_3OD, 175 MHz)……S23
Figure S21. ESIMS spectrum of **8**……S24
Figure S22. ^{1}H NMR spectrum of **9** ($CDCl_3$, 700 MHz)……S25
Figure S23. ESIMS spectrum of **9**……S26
Figure S24. ^{1}H NMR spectrum of **10** ($CDCl_3$, 700 MHz)……S27
Figure S25. ^{1}H NMR spectrum of **11** ($CDCl_3$, 700 MHz)……S28
Figure S26. ^{13}C NMR spectrum of **11** ($CDCl_3$, 175 MHz)……S29
Figure S27. ESIMS spectrum of **11**……S30
Figure S28. ^{1}H NMR spectrum of **12** ($CDCl_3$, 700 MHz)……S31
Figure S29. ^{1}H NMR spectrum of **13** ($CDCl_3$, 700 MHz)……S32
Figure S30. ^{1}H NMR spectrum of **14** ($CDCl_3$, 700 MHz)……S33

Figure S31. ^{13}C NMR spectrum of **14** ($CDCl_3$, 175 MHz)……….........................……………………………………………………………S34
In Vitro Cytotoxicity Test…………………………………………………………………………………………………...…………. S35

Figure S1. ^{1}H NMR spectrum of **1** (CD_3OD, 700 MHz)

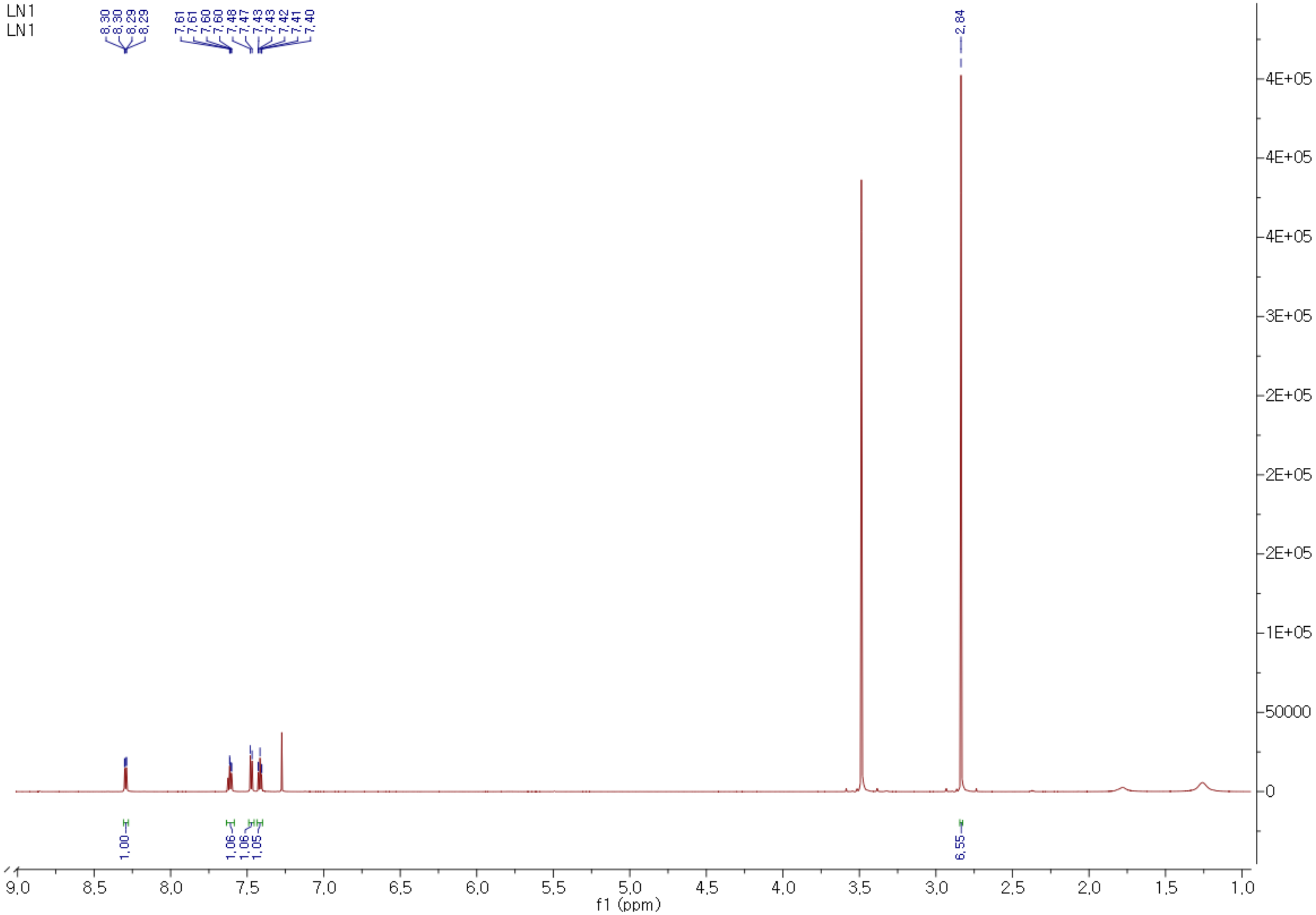

Figure S2. ^{13}C NMR spectrum of **1** (CD_3OD, 175 Hz)

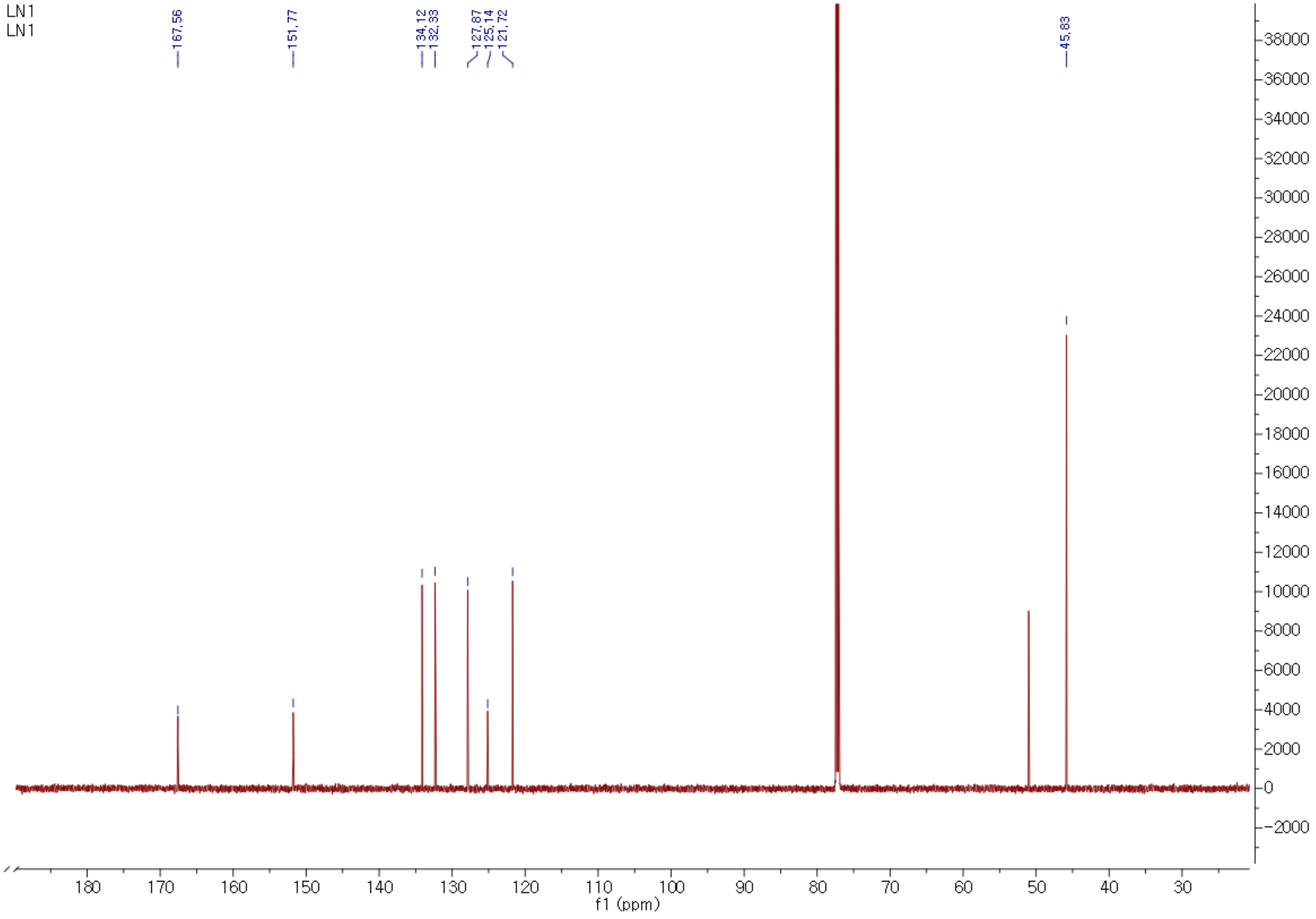

Figure S3. ESIMS spectrum of **1**

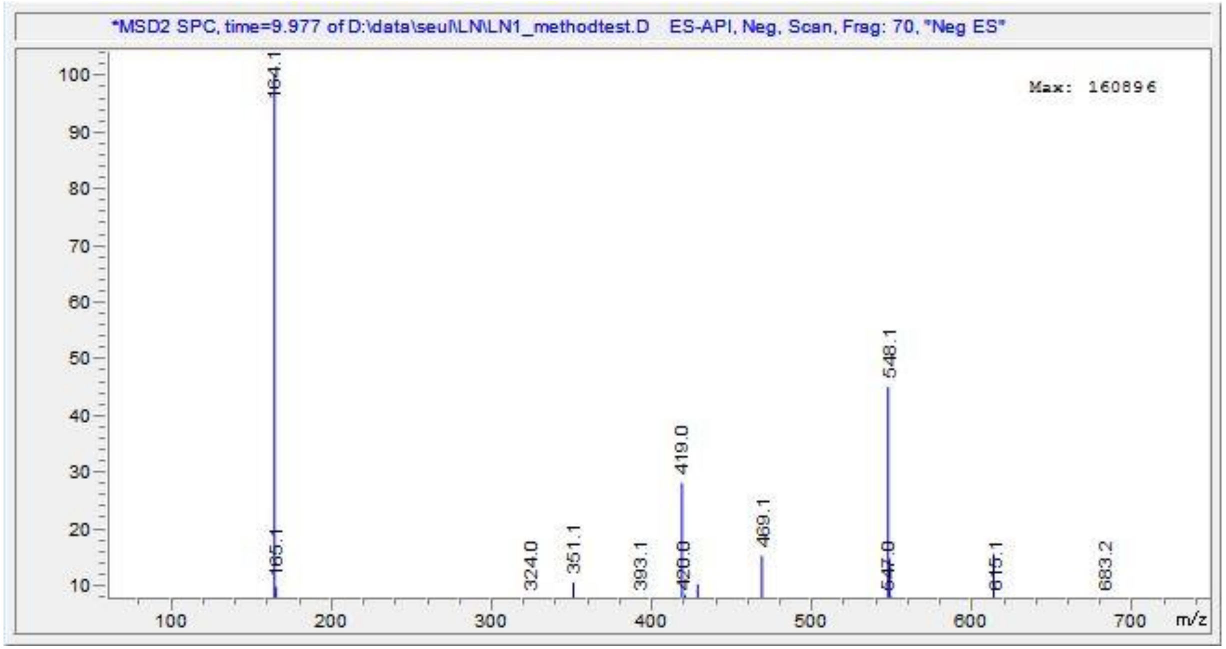

Figure S4. ^{1}H NMR spectrum of **2** (CD_3OD, 700 MHz)

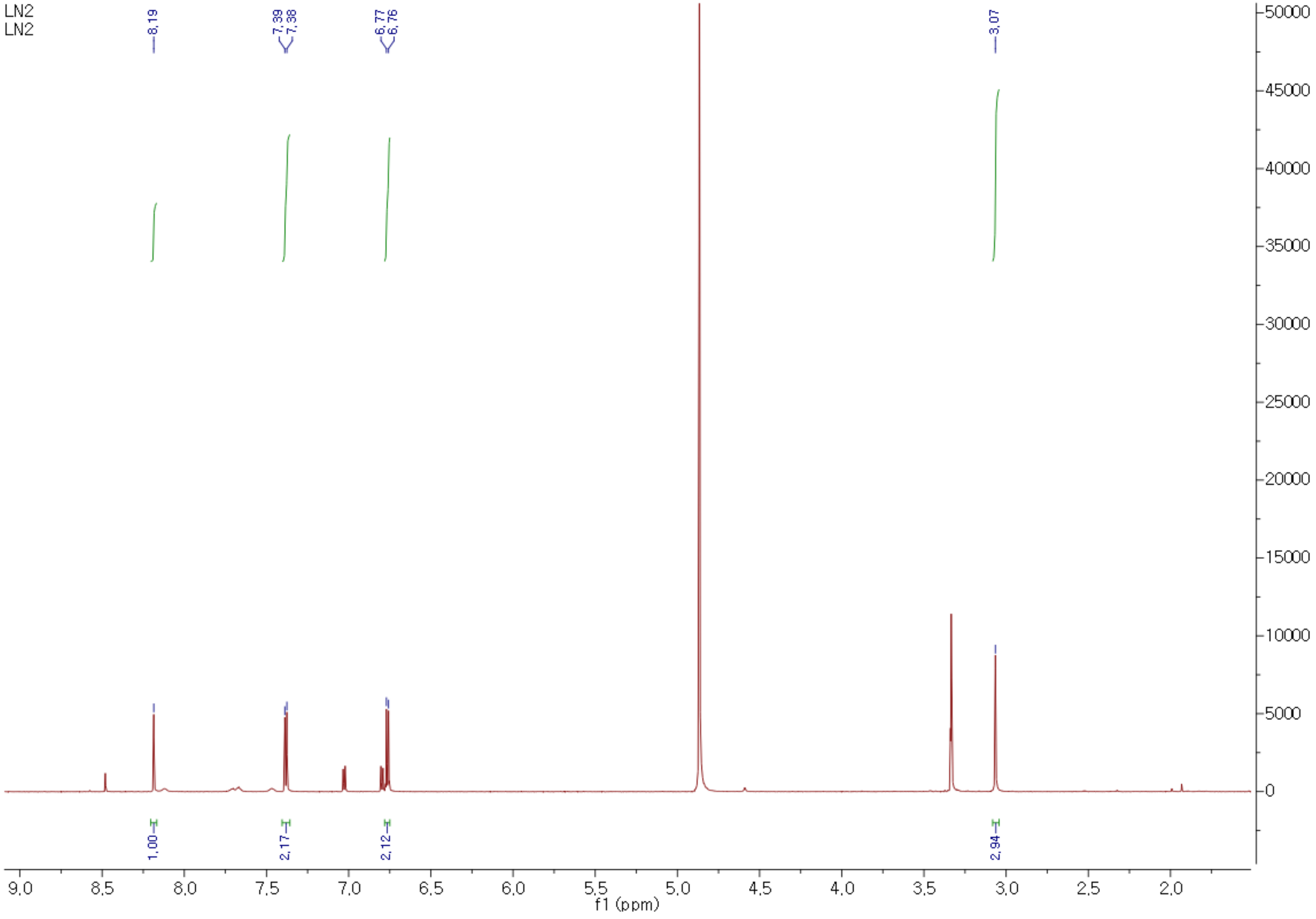

Figure S5. ^{13}C NMR spectrum of **2** (CD_3OD, 175 MHz)

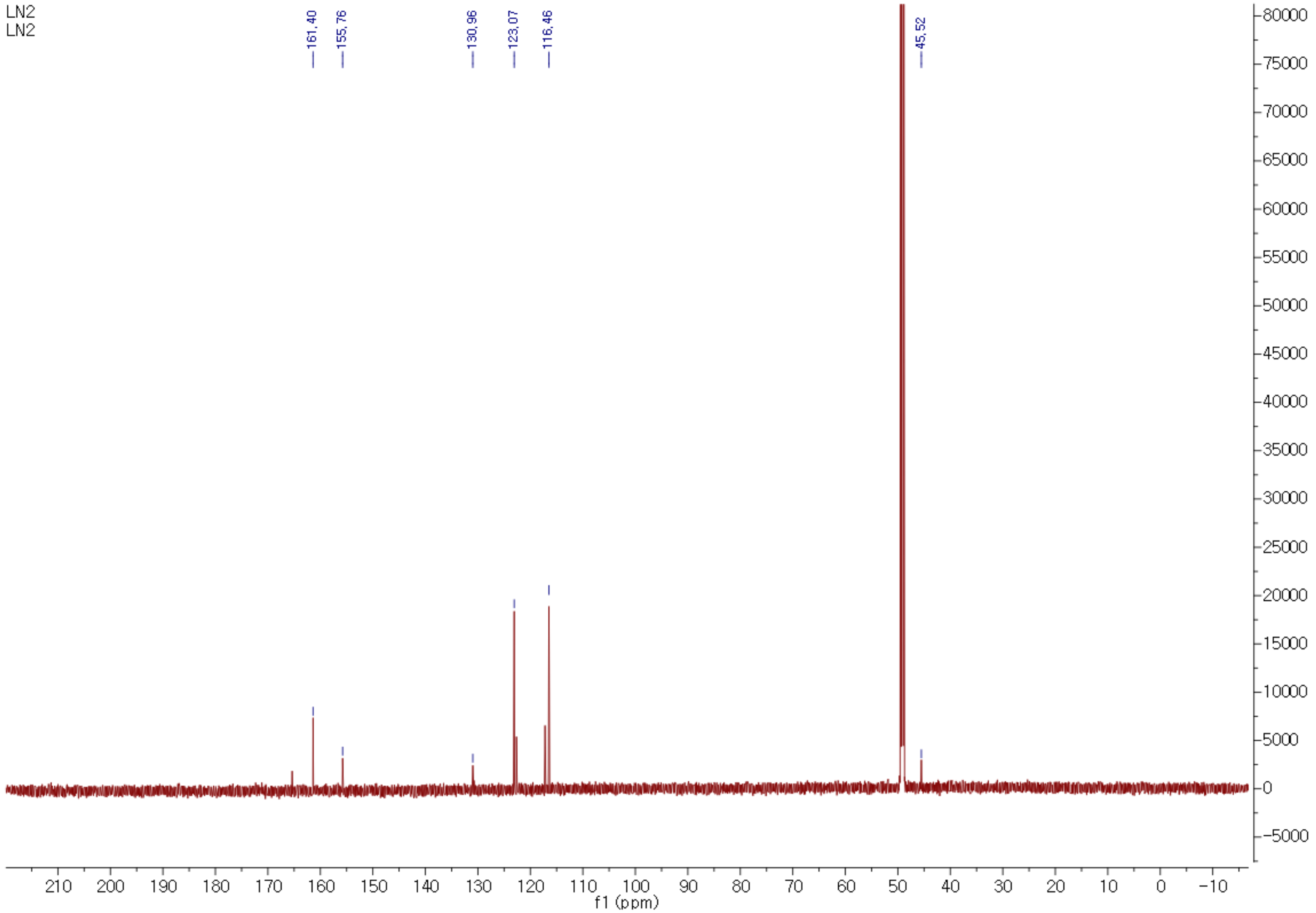

Figure S6. ^{1}H NMR spectrum of **3** (CD_3OD, 700 MHz)

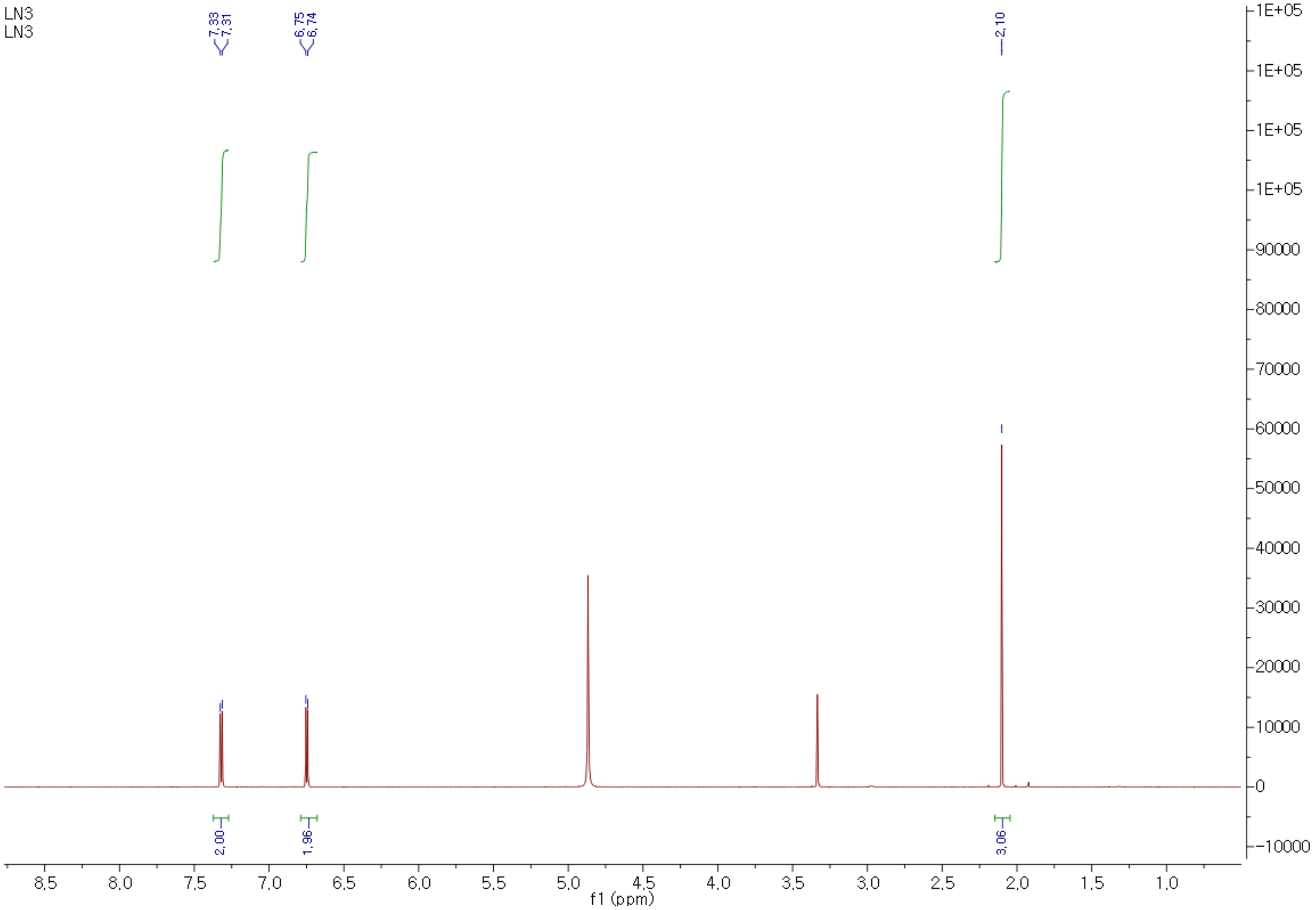

Figure S7. ^{13}C NMR spectrum of **3** (CD_3OD, 175 MHz)

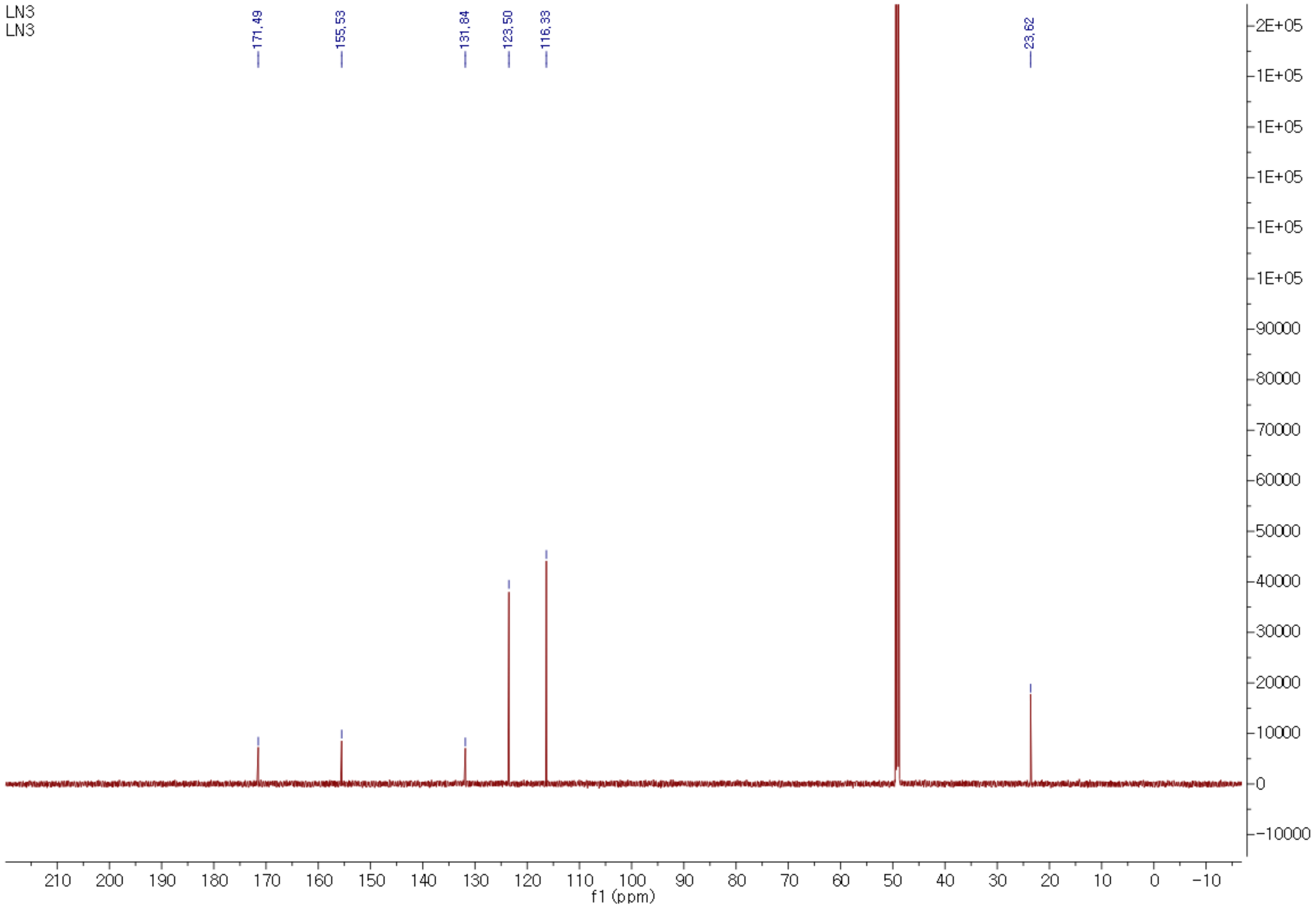

Figure S8. ESIMS spectrum of **3**

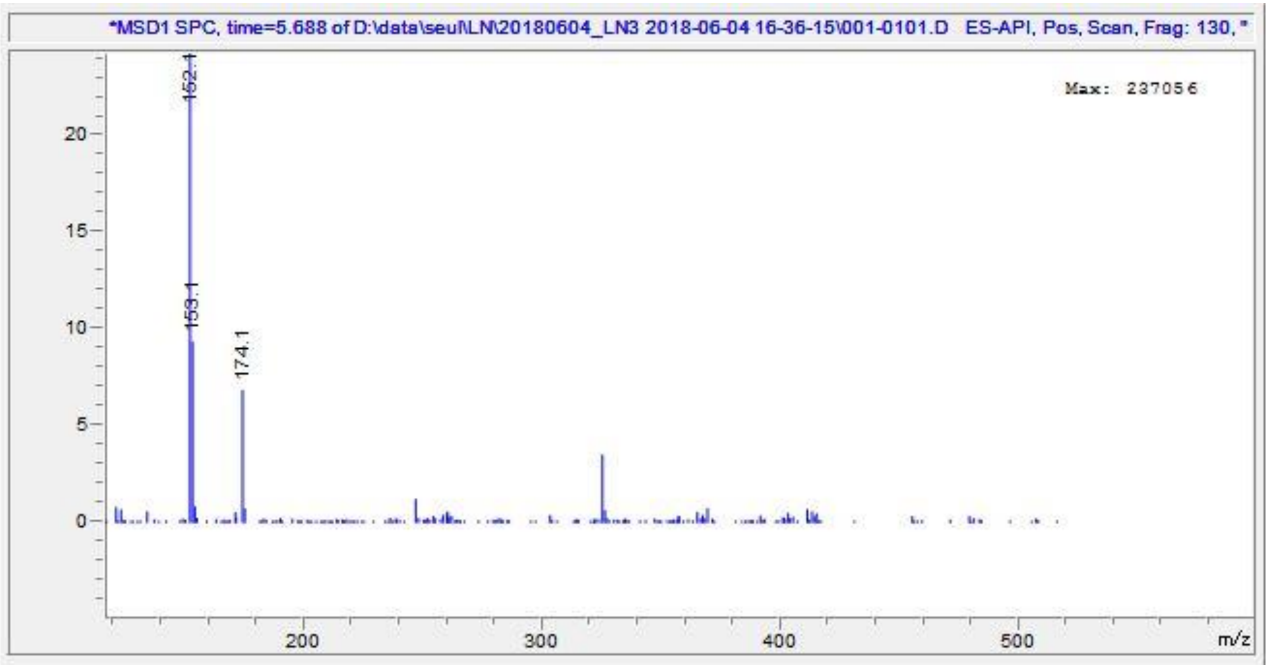

Figure S9. ^{1}H NMR spectrum of **4** (CD_3OD, 700 MHz)

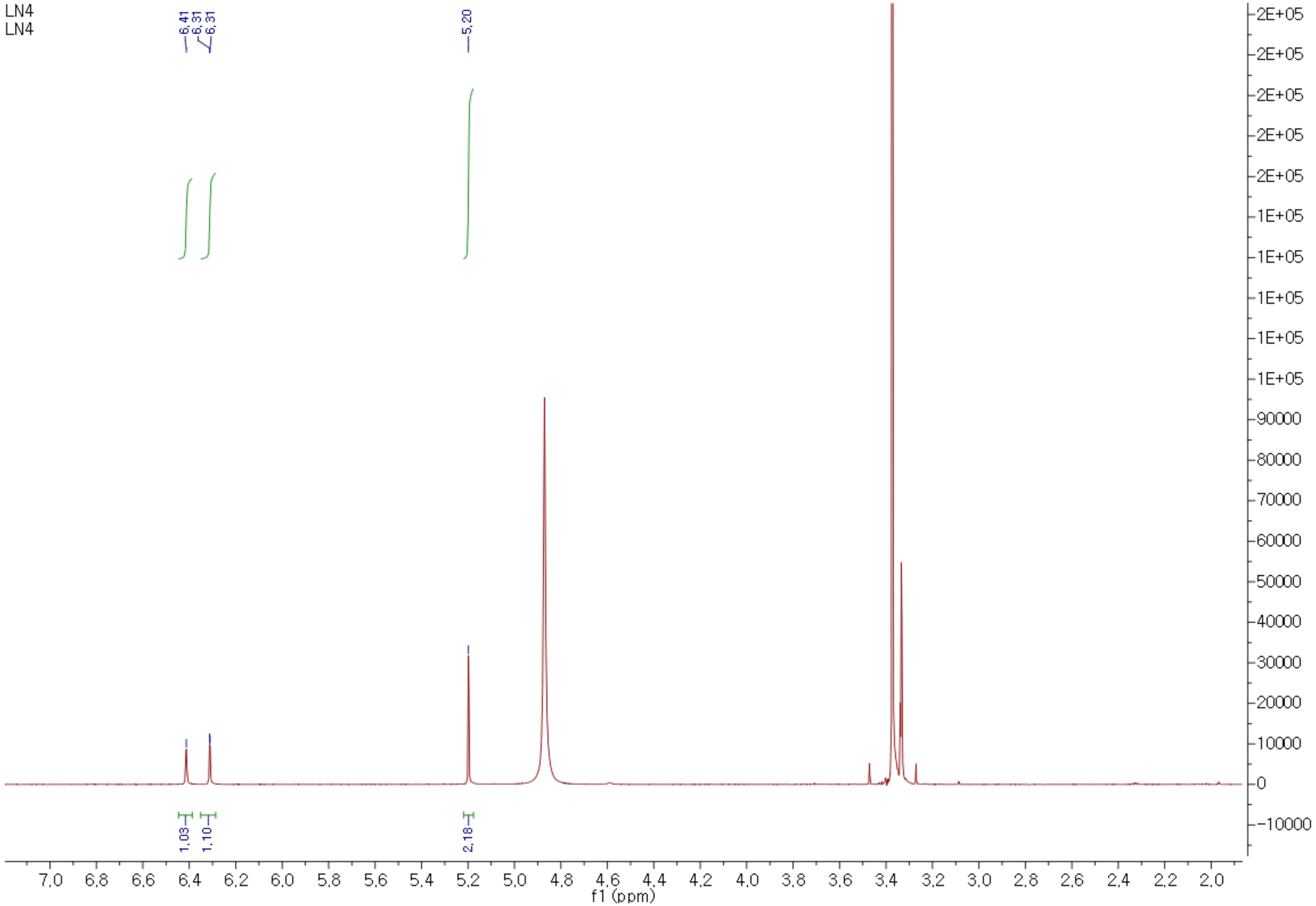

Figure S10. ^{13}C NMR spectrum of **4** (CD_3OD, 175 MHz)

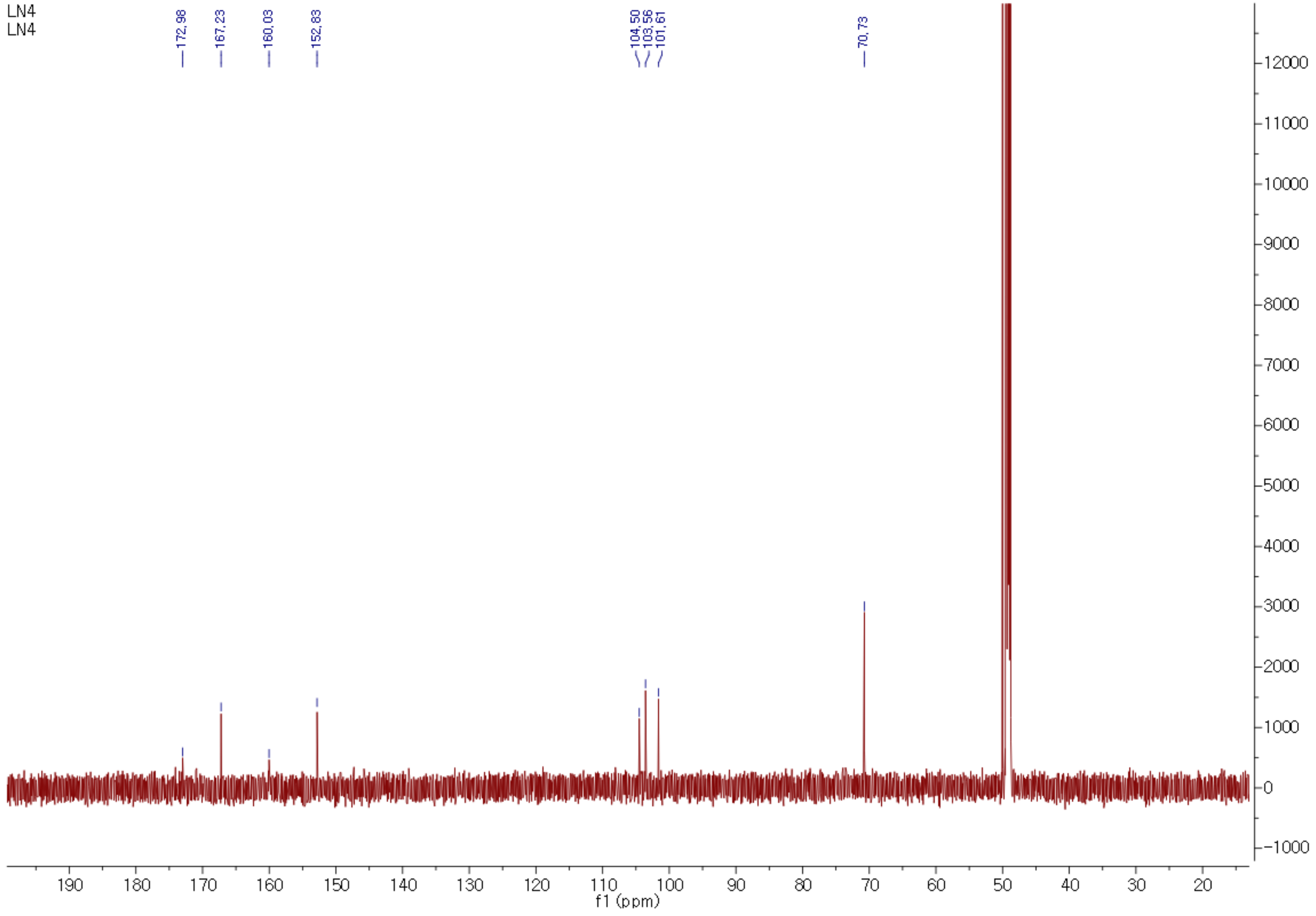

Figure S11. ESIMS spectrum of **4**

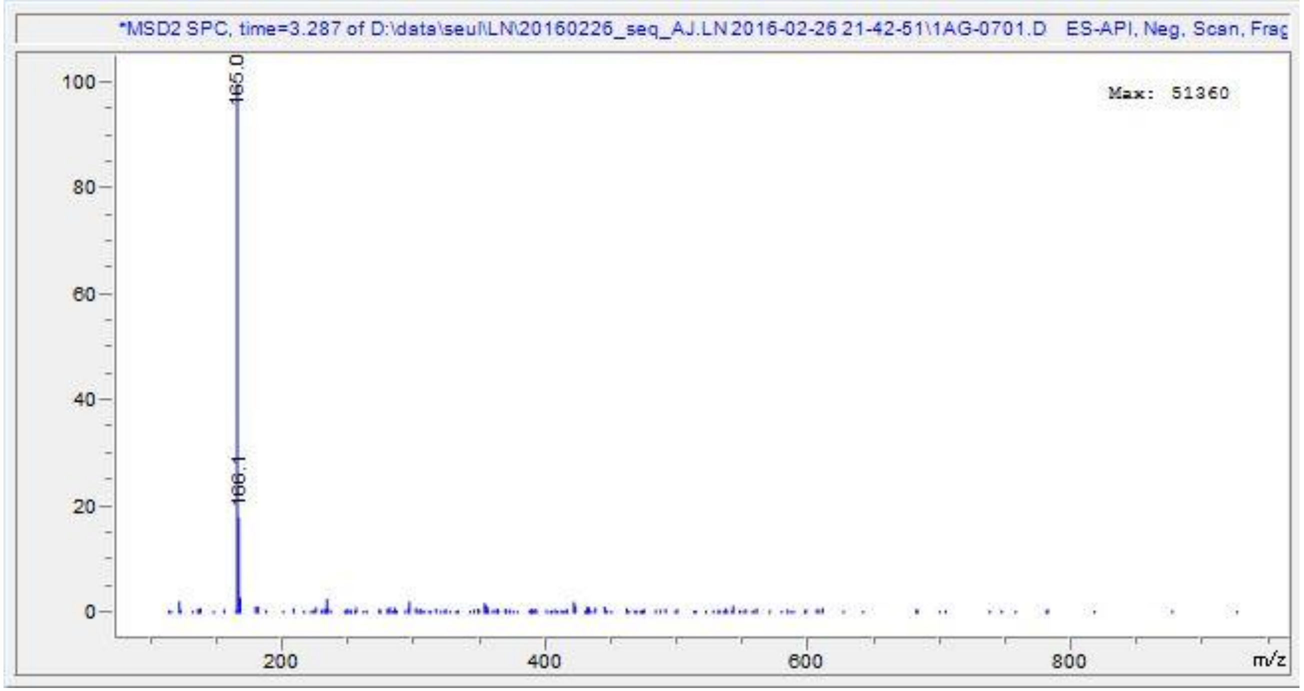

Figure S12. ^{1}H NMR spectrum of **5** ($CDCl_3$, 700 MHz)

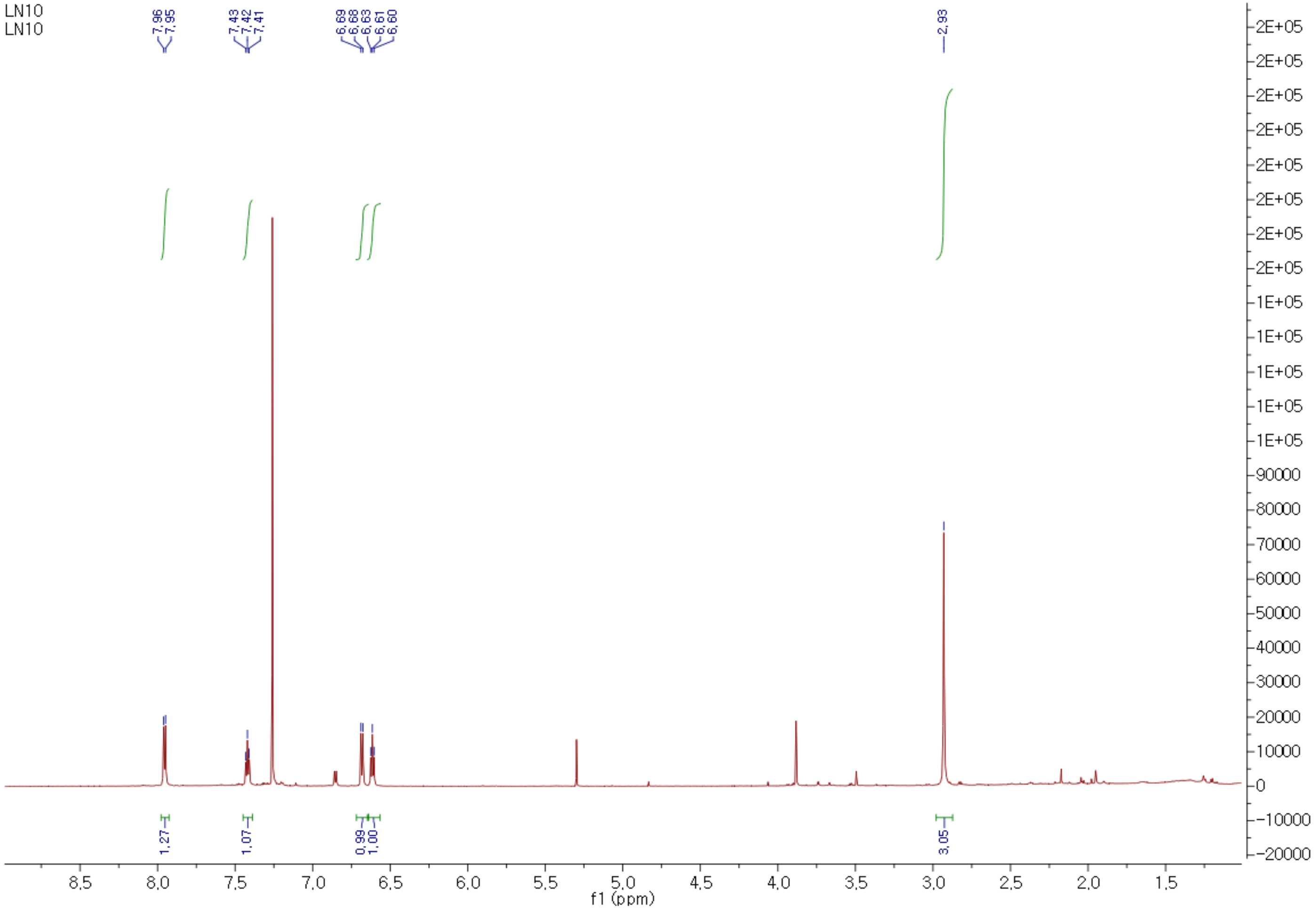

Figure S13. ^{13}C NMR spectrum of **5** ($CDCl_3$, 175 MHz)

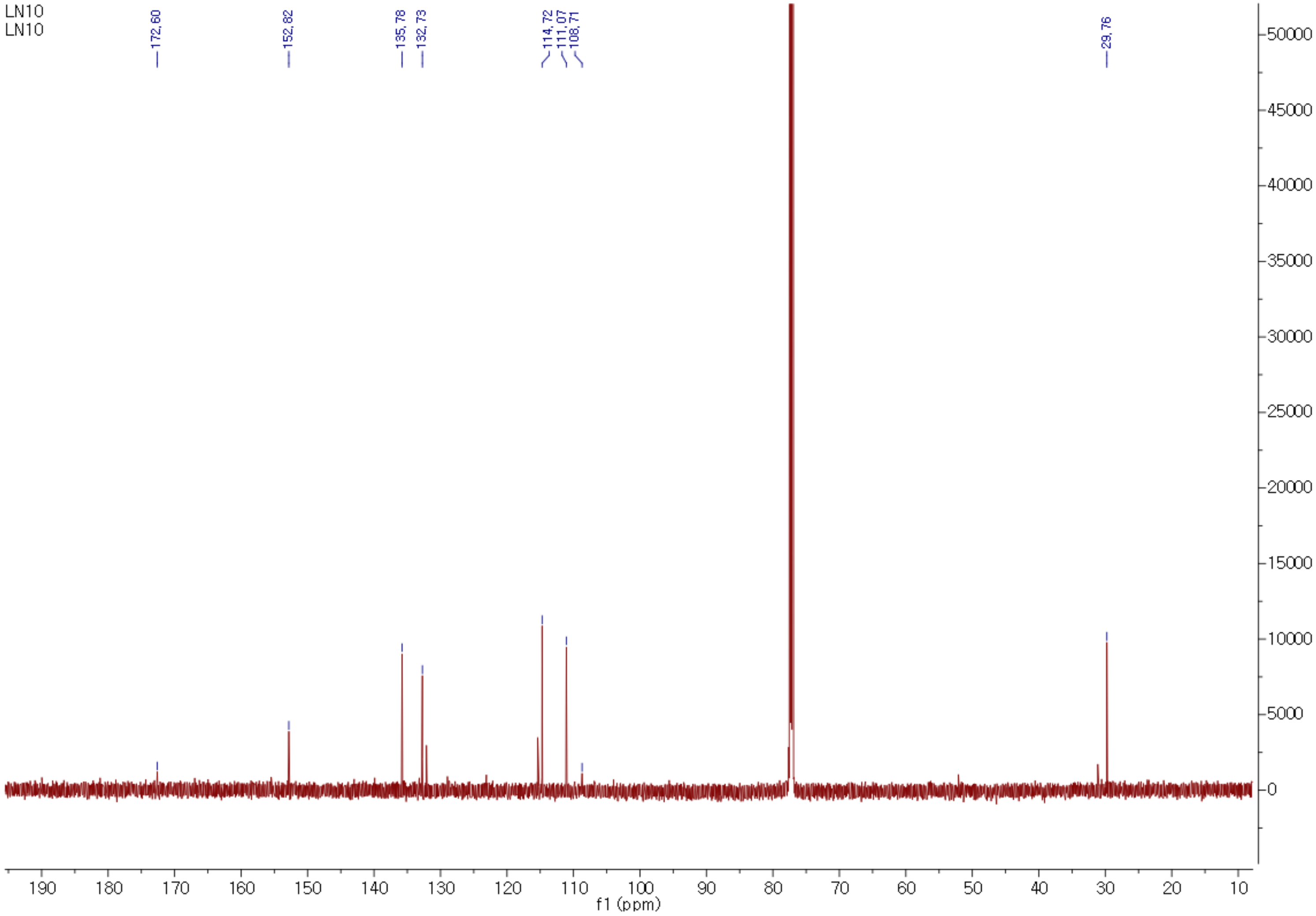

Figure S14. ESIMS spectrum of **5**

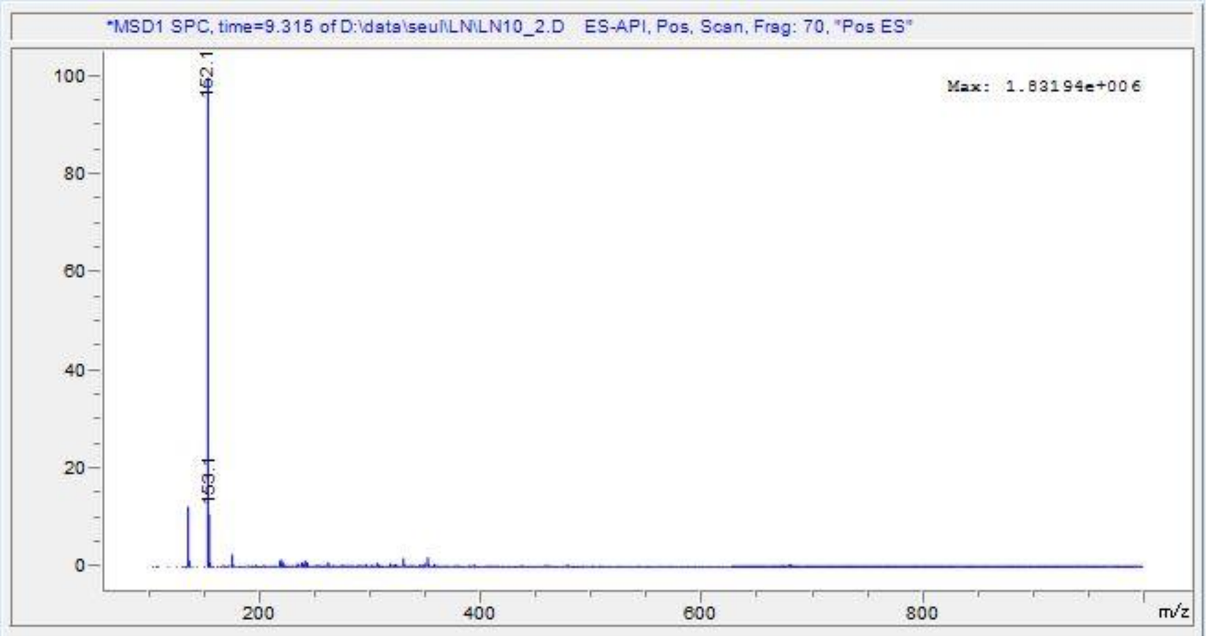

Figure S15. ^{1}H NMR spectrum of **6** (CD_3OD, 700 MHz)

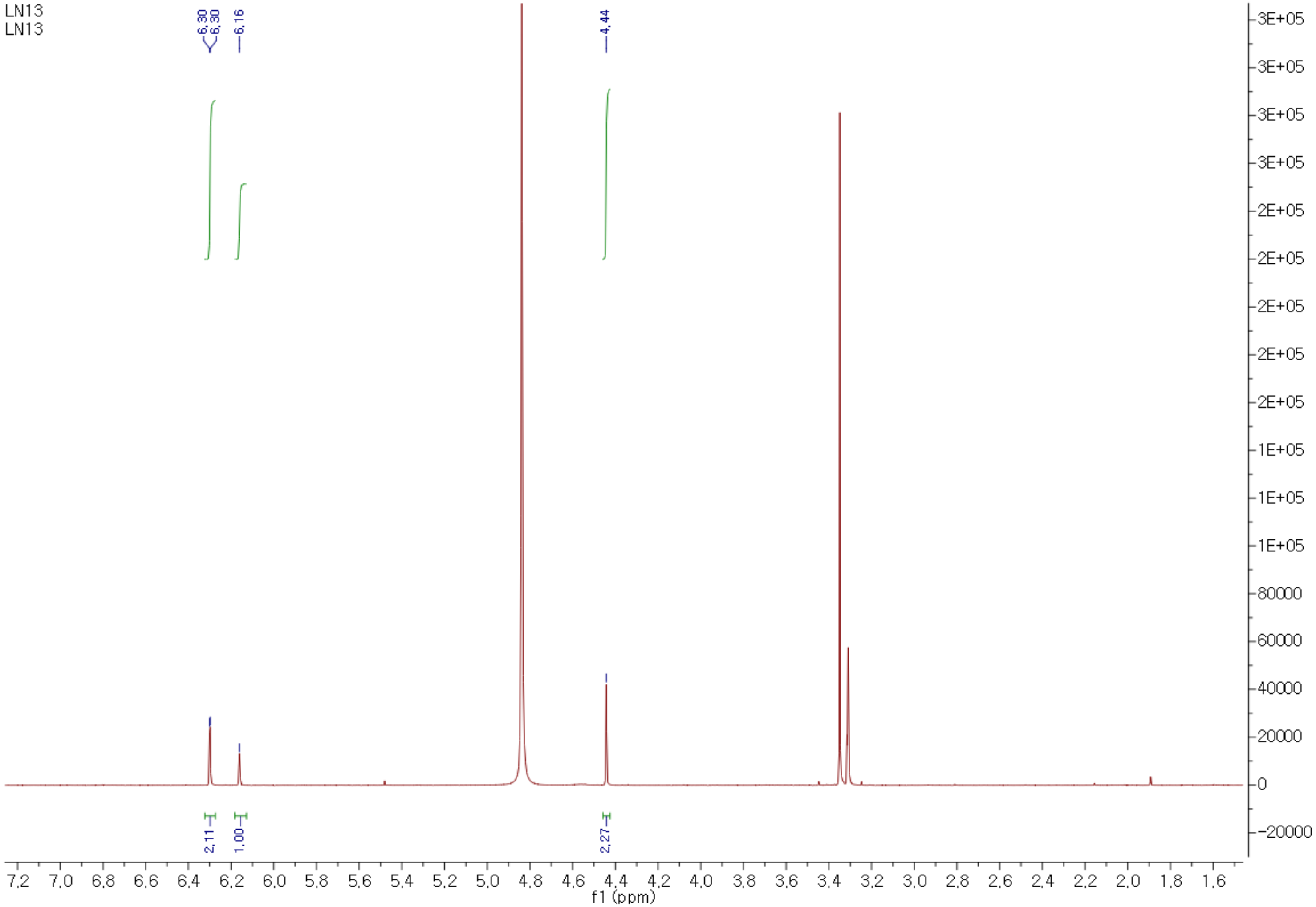

Figure S16. ESIMS spectrum of **6**

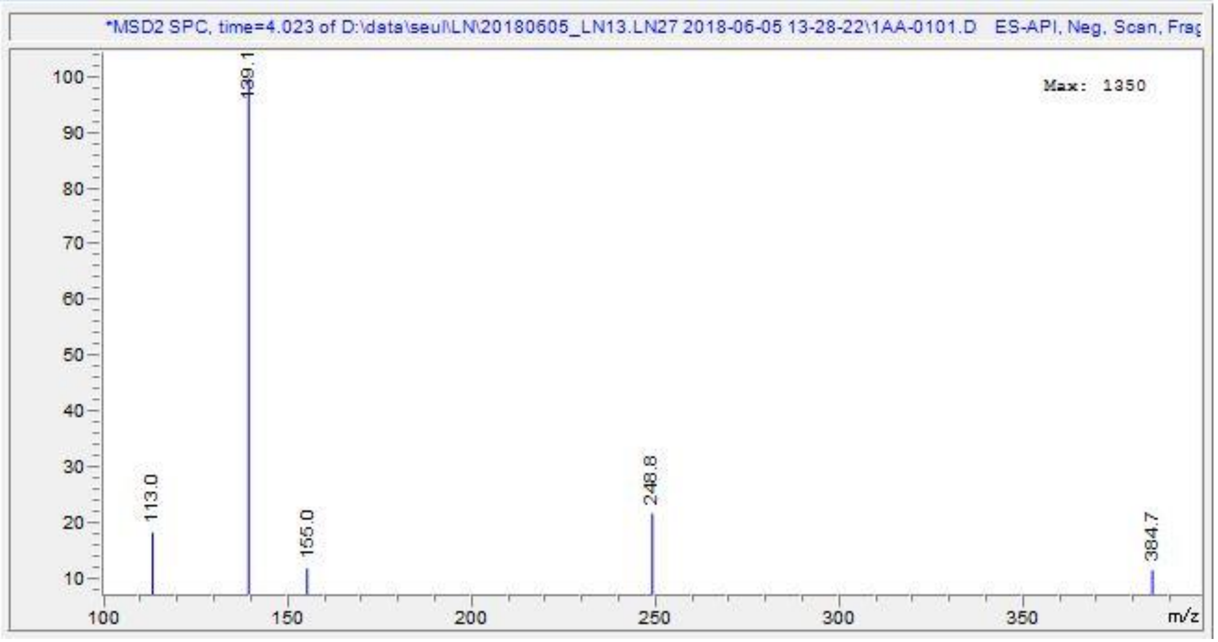

Figure S17. ^{1}H NMR spectrum of **7** ($CDCl_3$, 700 MHz)

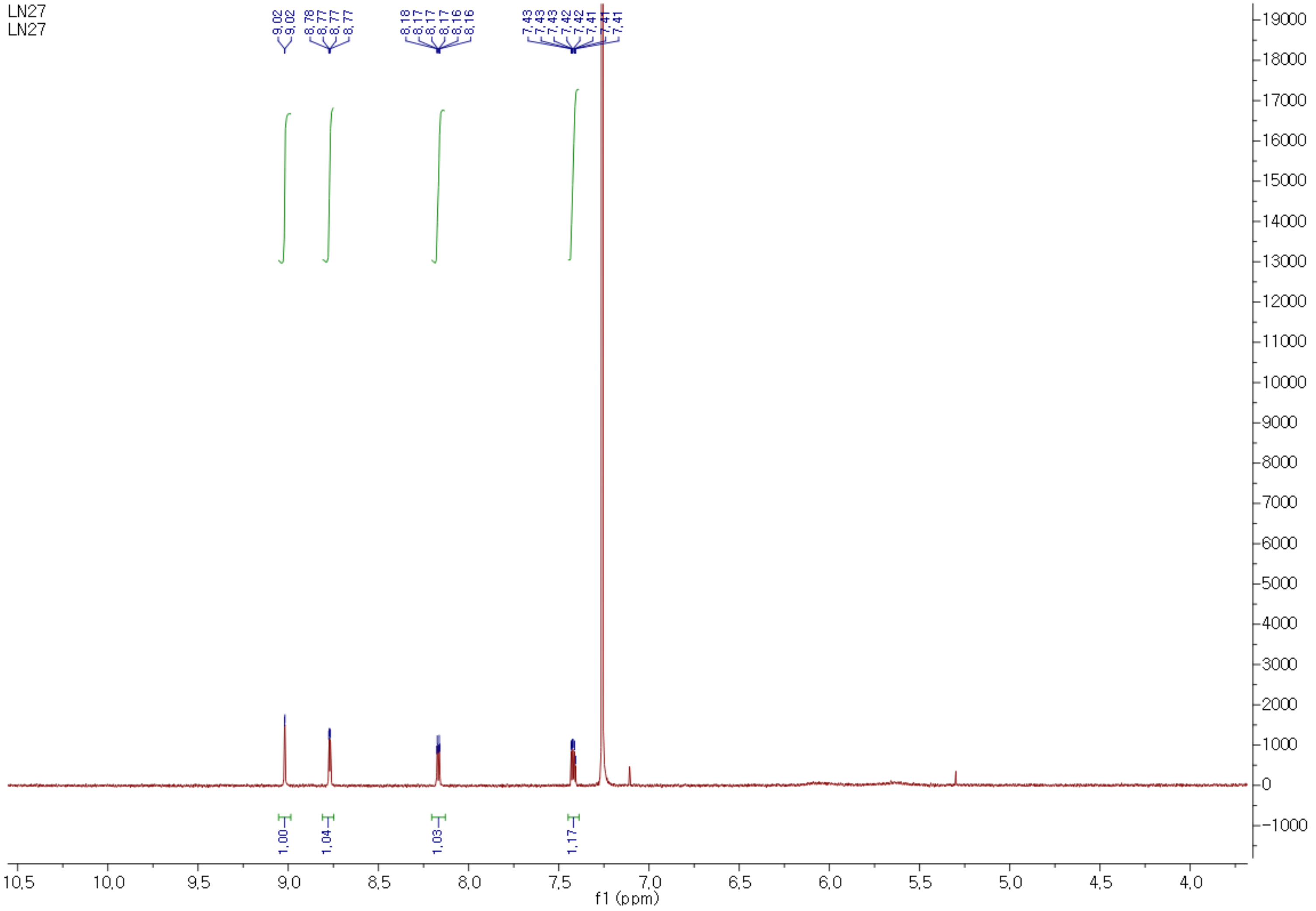

Figure S18. ESIMS spectrum of **7**

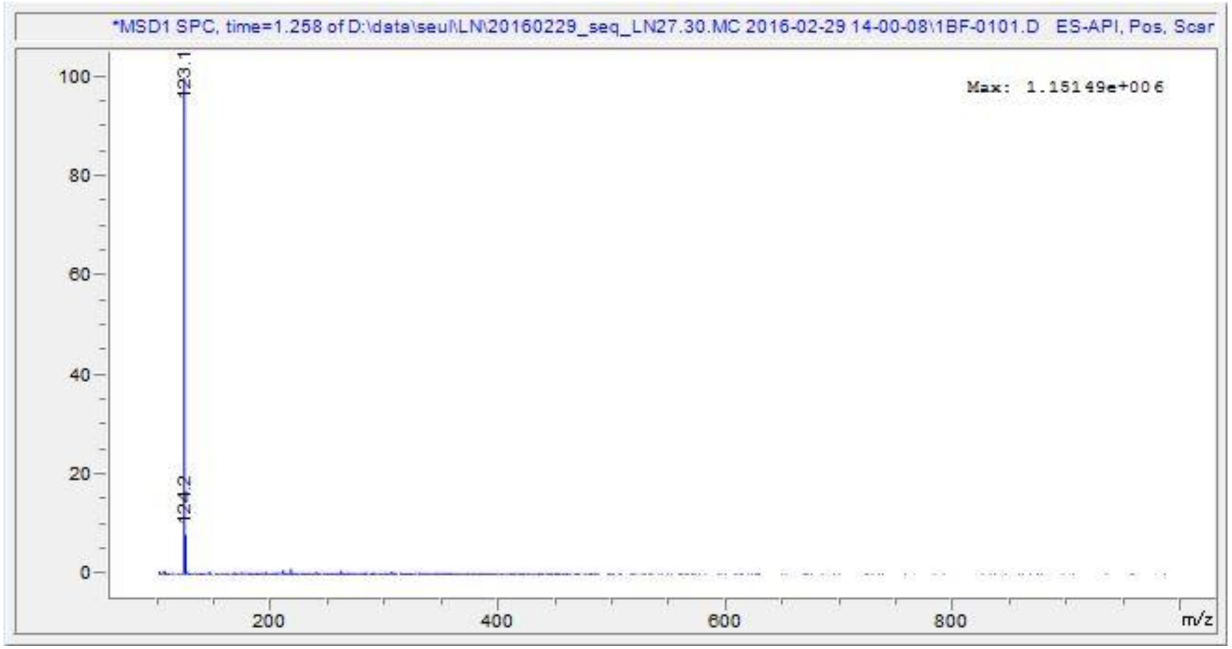

Figure S19. ^{1}H NMR spectrum of **8** (CD_3OD, 700 MHz)

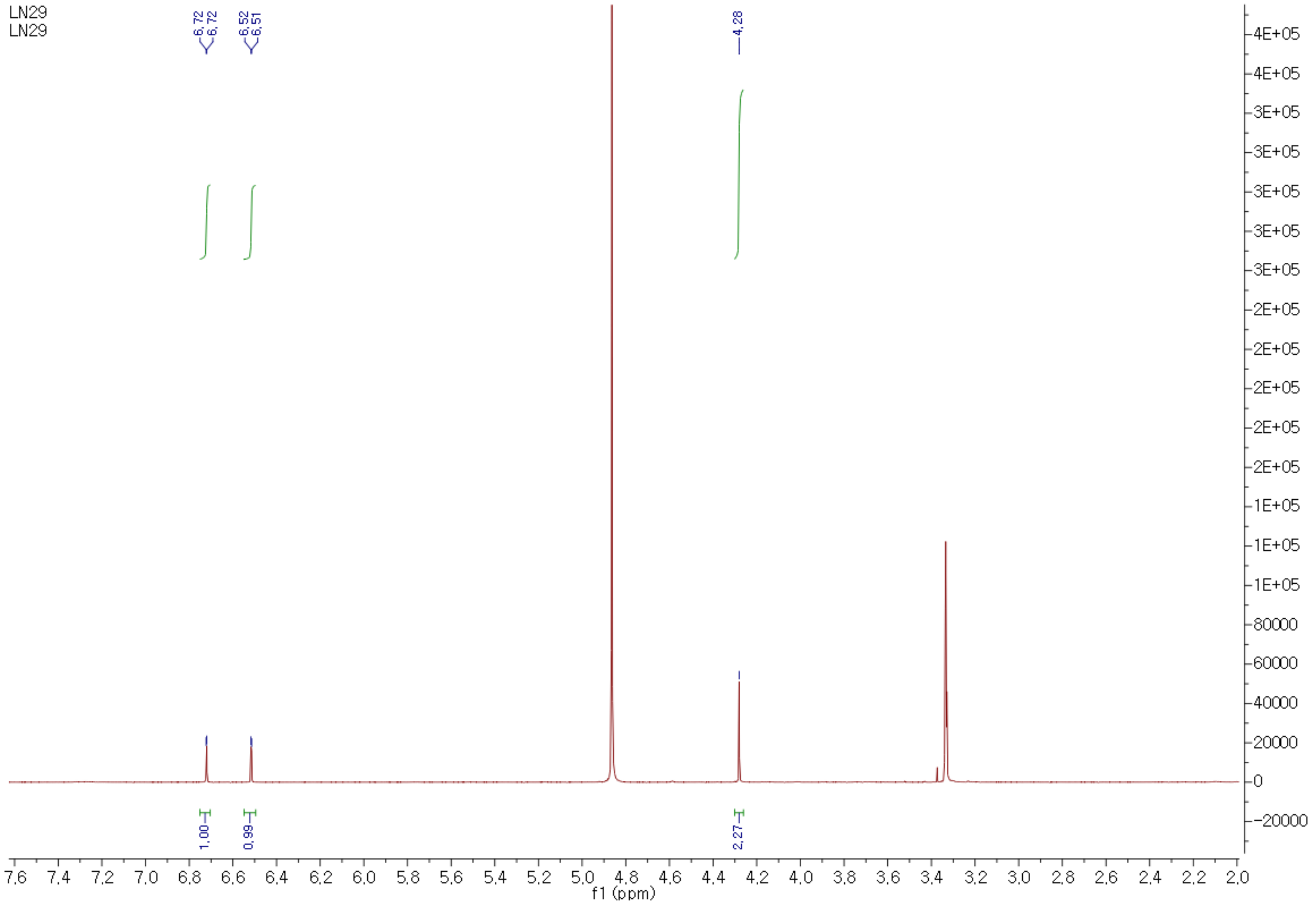

Figure S20. ^{13}C NMR spectrum of **8** (CD_3OD, 175 MHz)

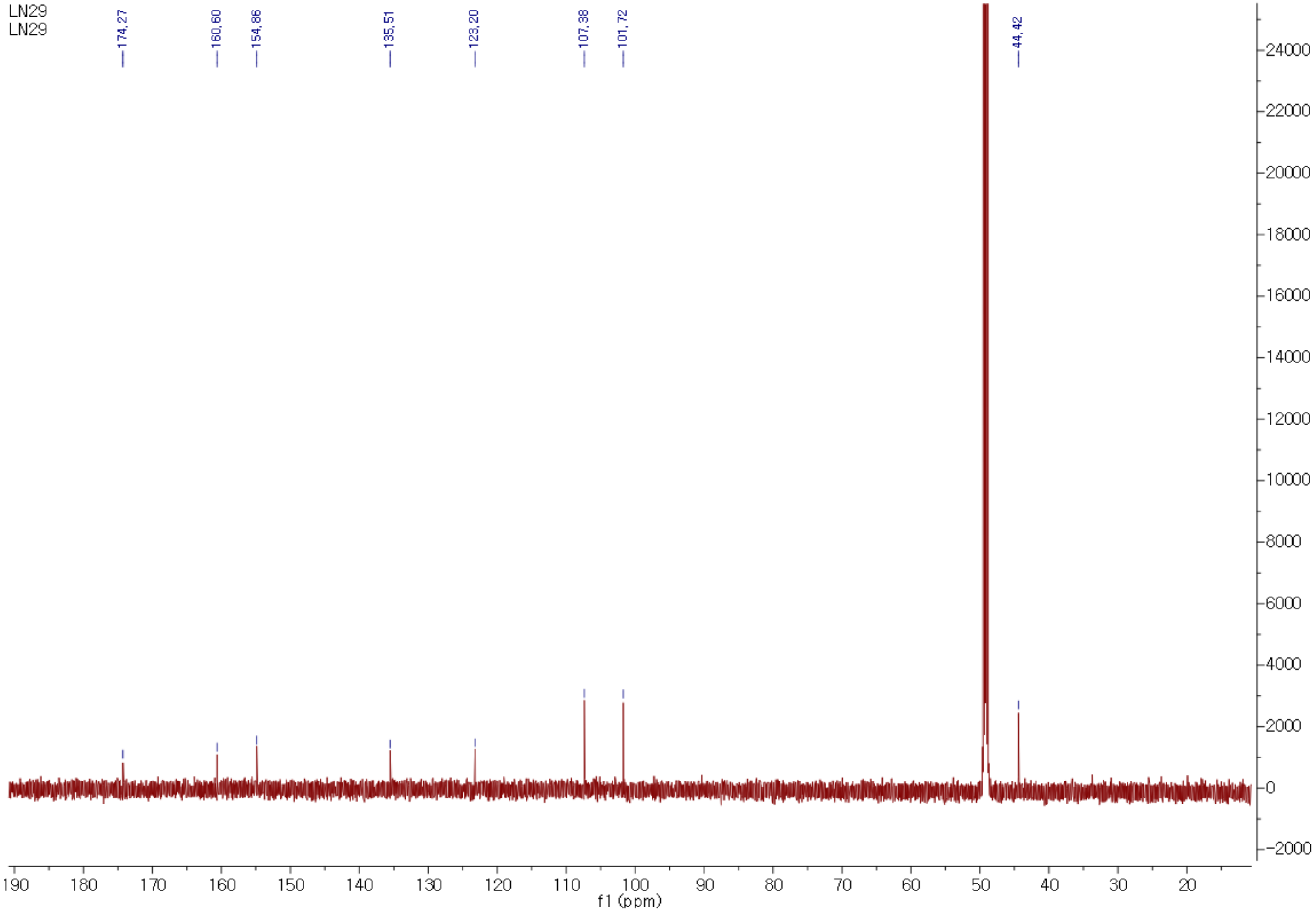

Figure S21. ESIMS spectrum of **8**

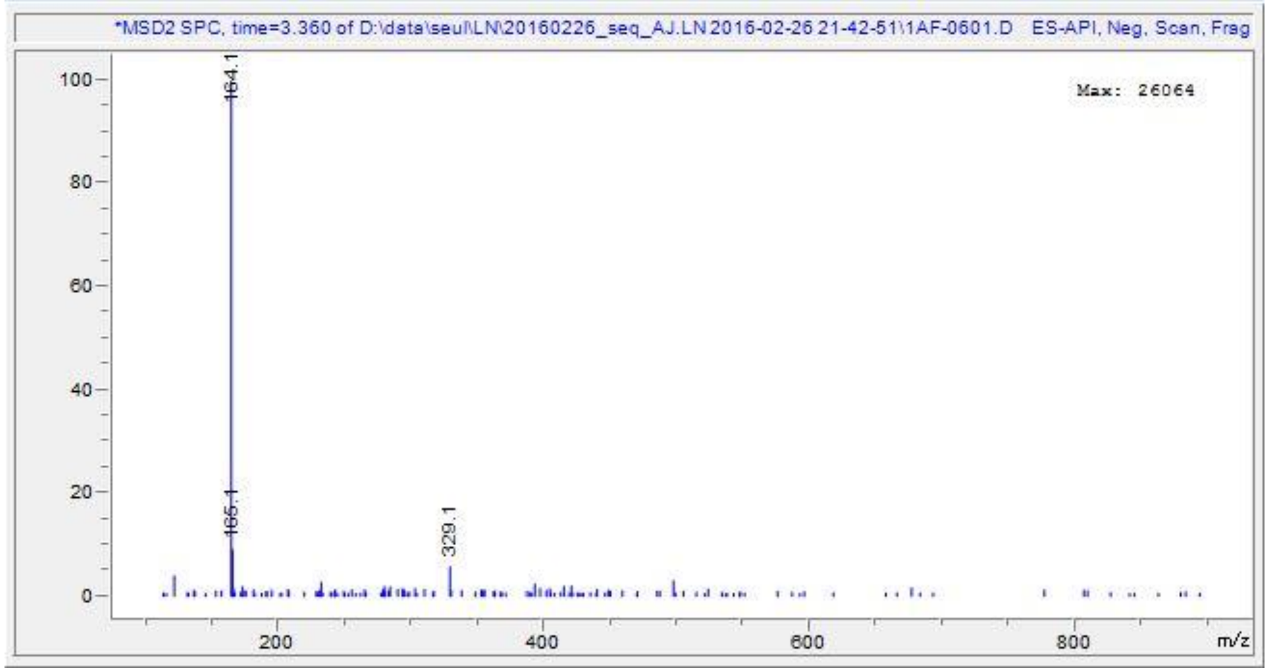

Figure S22. ^{1}H NMR spectrum of **9** ($CDCl_3$, 700 MHz)

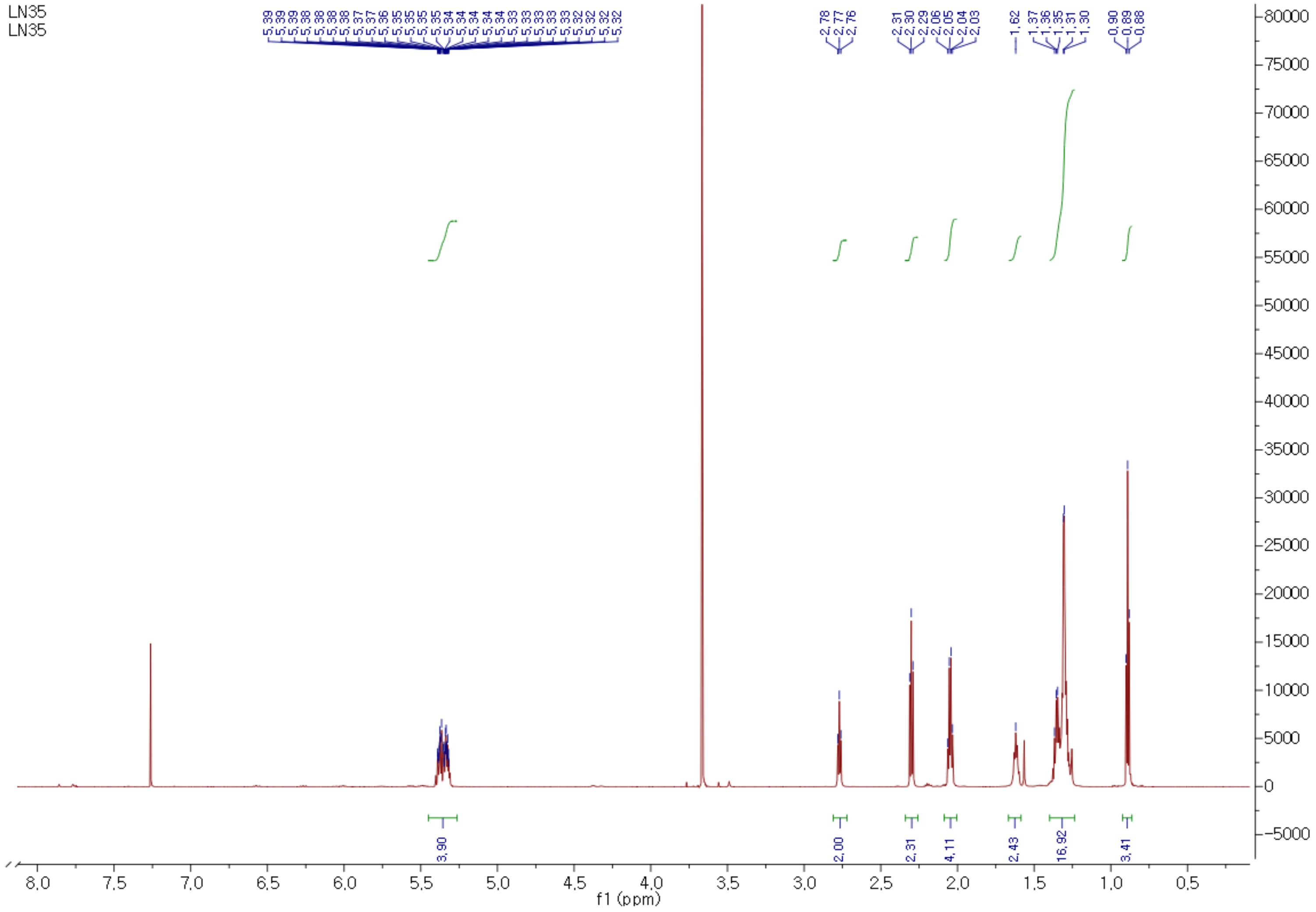

Figure S23. ESIMS spectrum of **9**

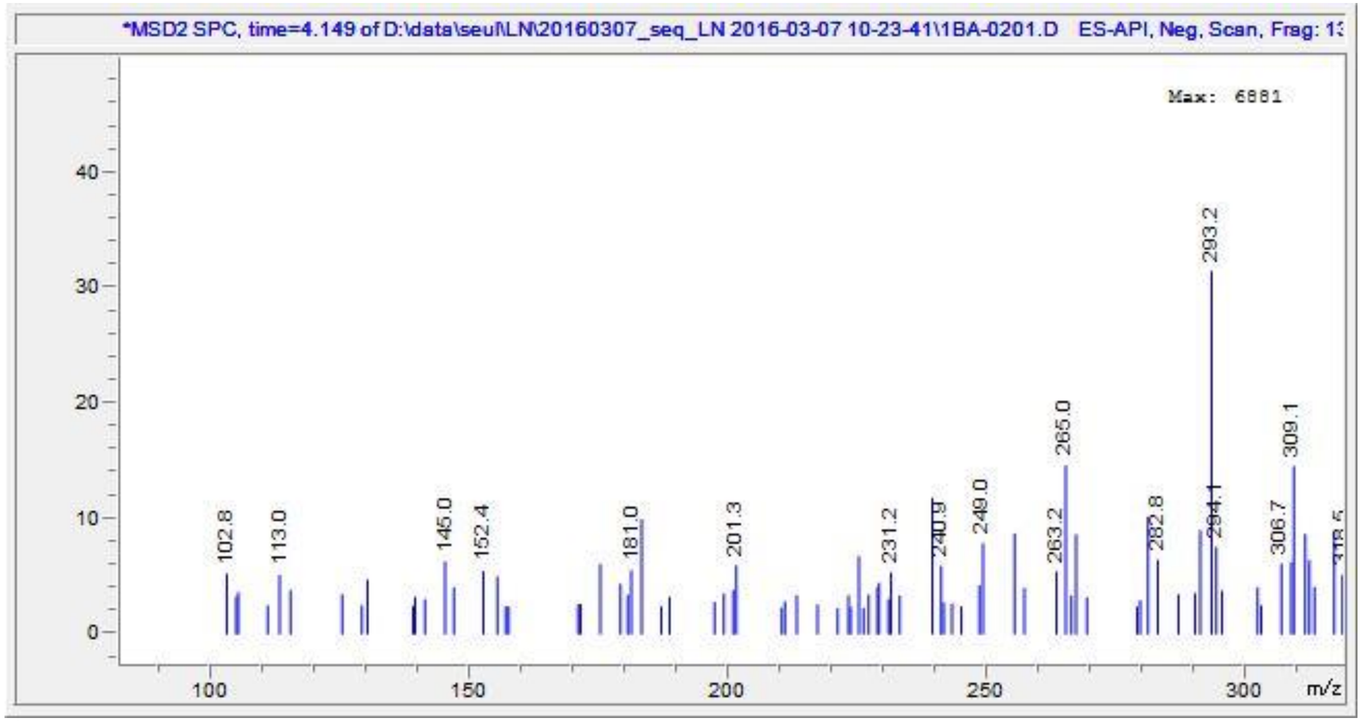

Figure S24. ^{1}H NMR spectrum of **10** ($CDCl_3$, 700 MHz)

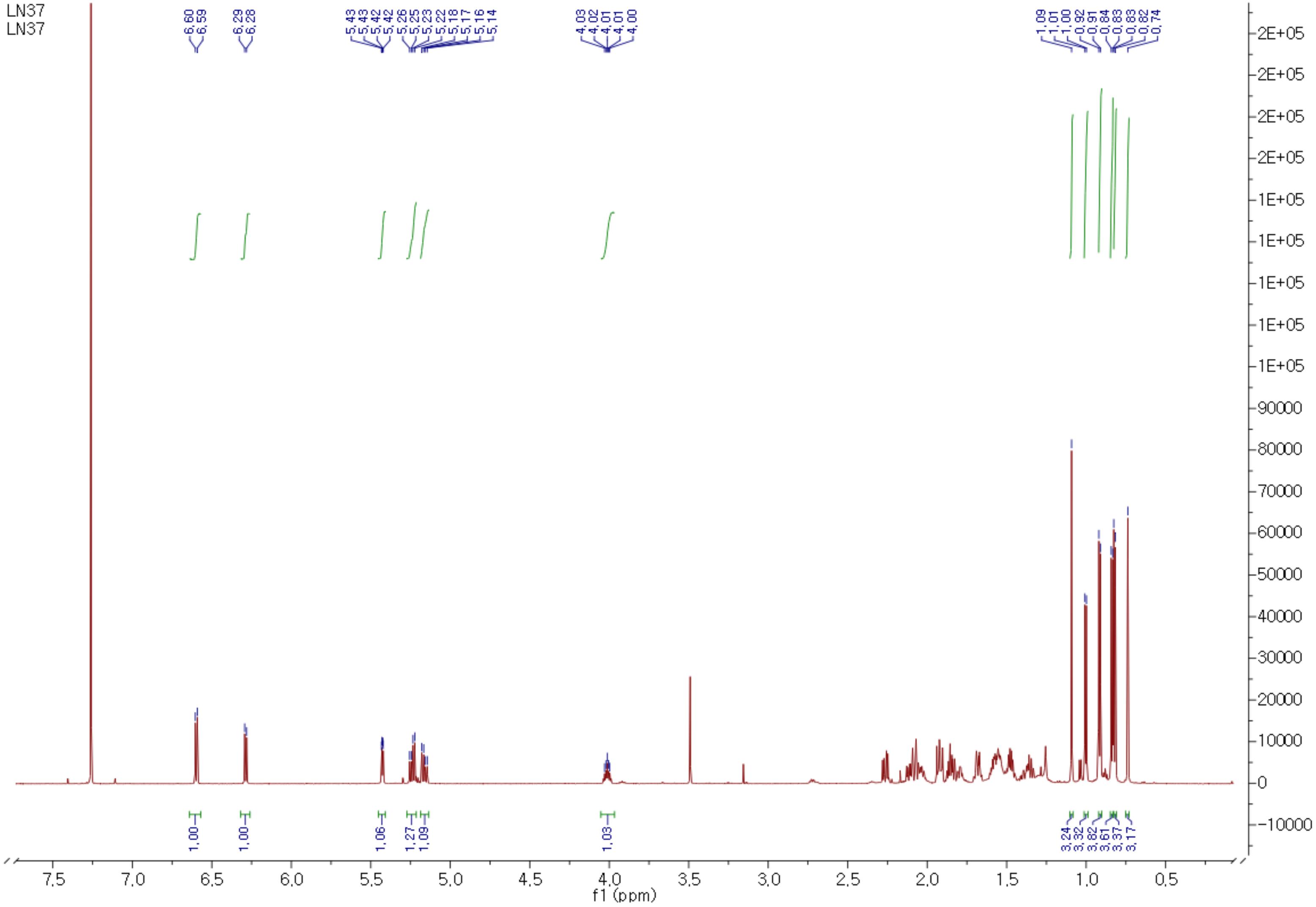

Figure S25. ^{1}H NMR spectrum of **11** ($CDCl_3$, 700 MHz)

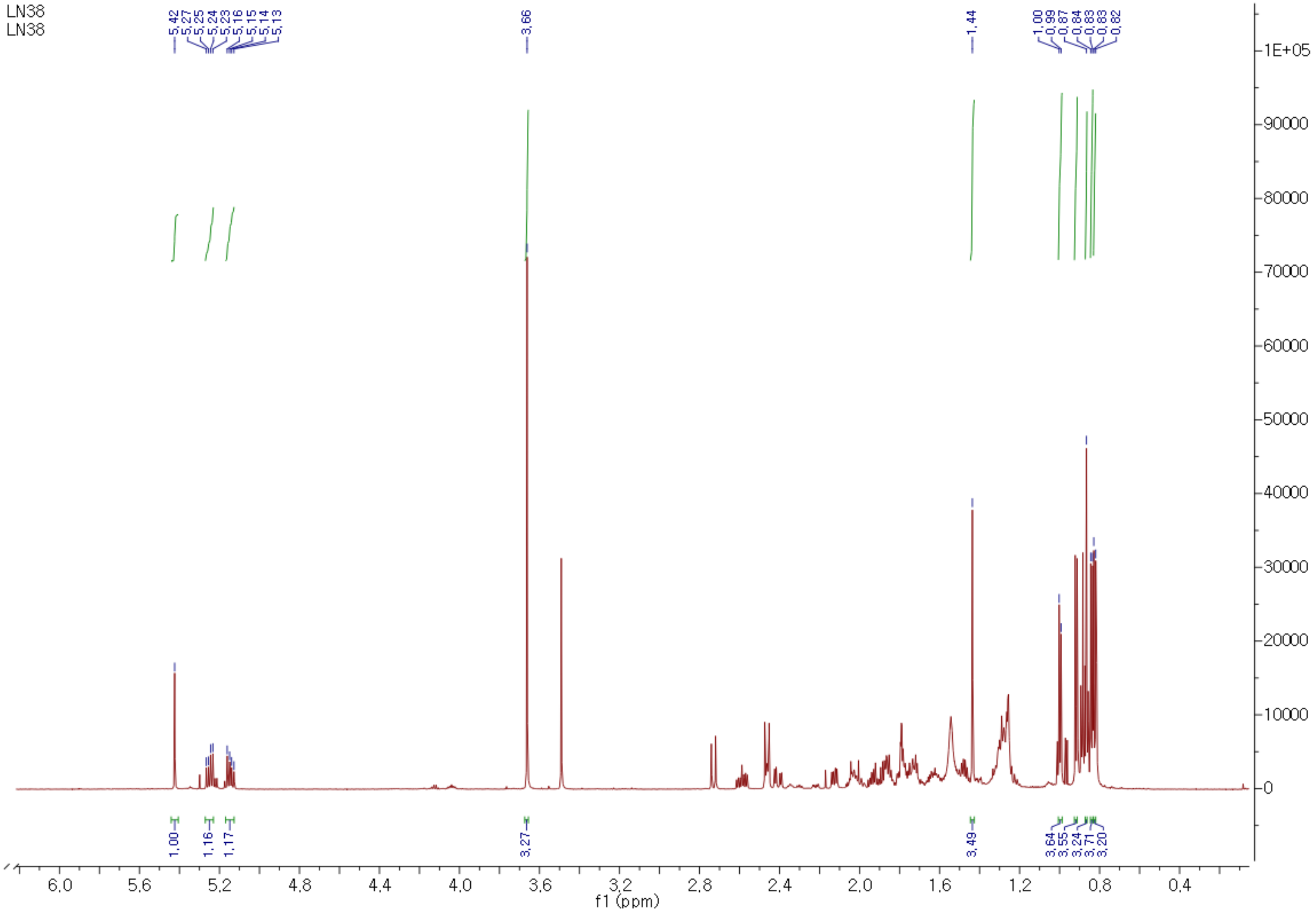

Figure S26. ^{13}C NMR spectrum of **11** ($CDCl_3$, 175 MHz)

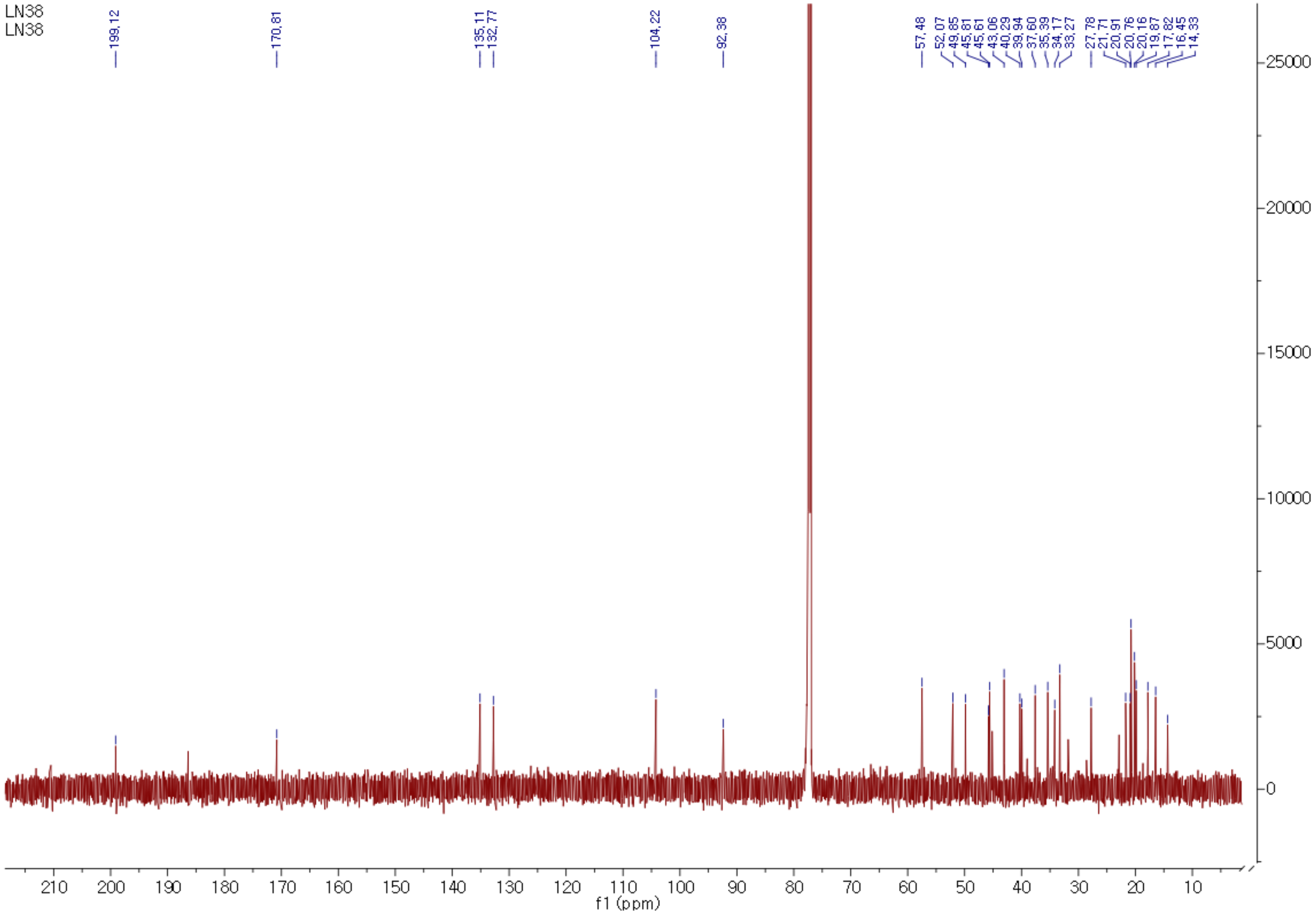

Figure S27. ESIMS spectrum of **11**

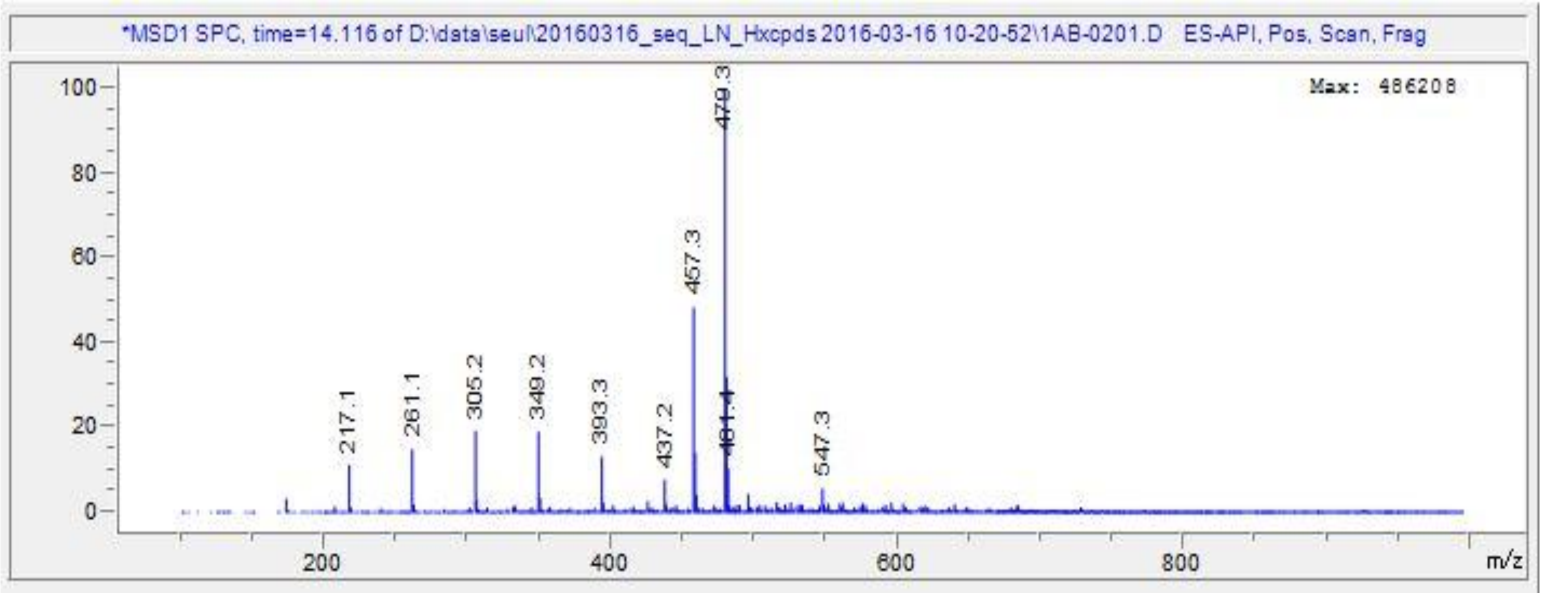

Figure S28. ^{1}H NMR spectrum of **12** ($CDCl_3$, 700 MHz)

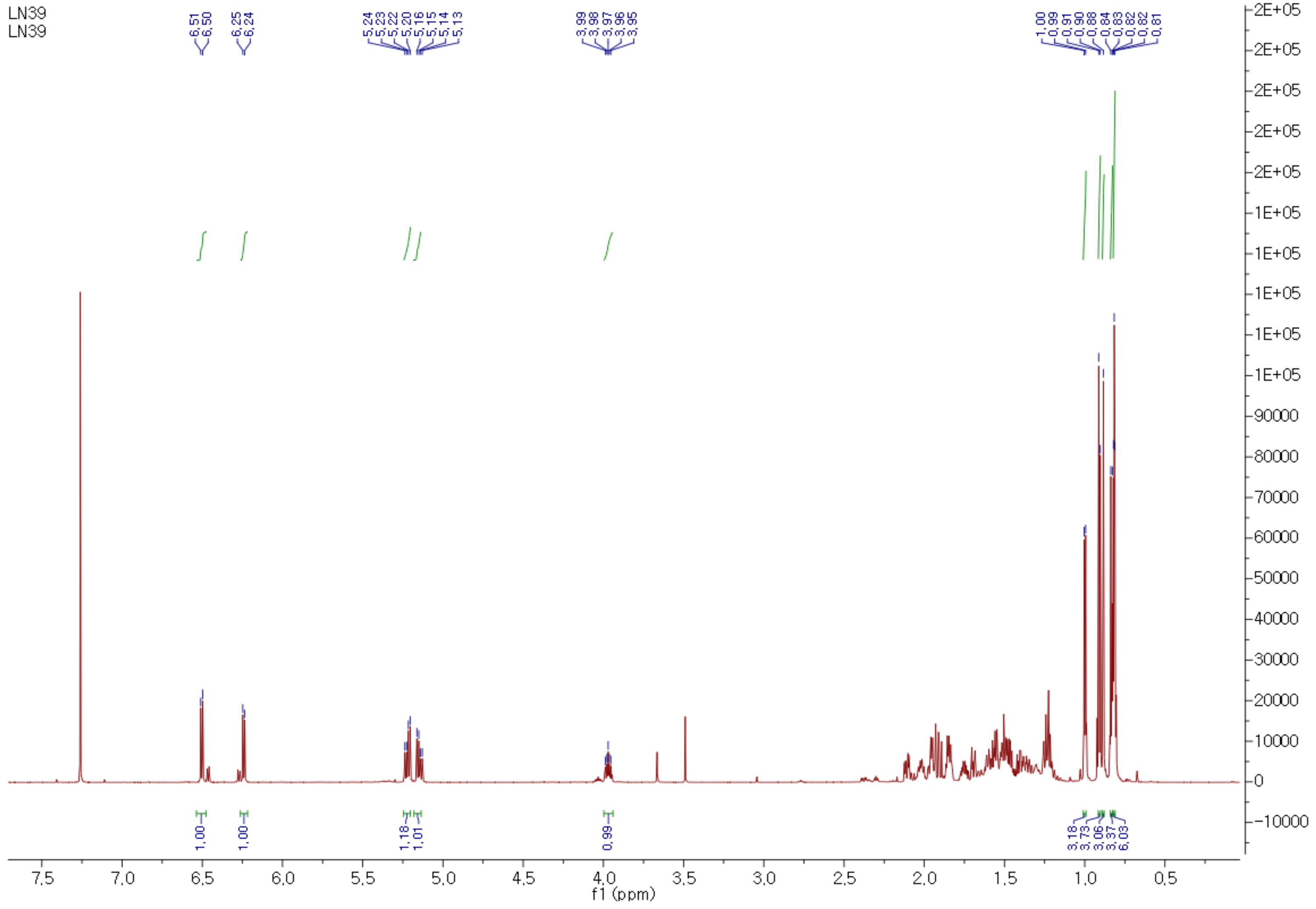

Figure S29. ^{1}H NMR spectrum of **13** ($CDCl_3$, 700 MHz)

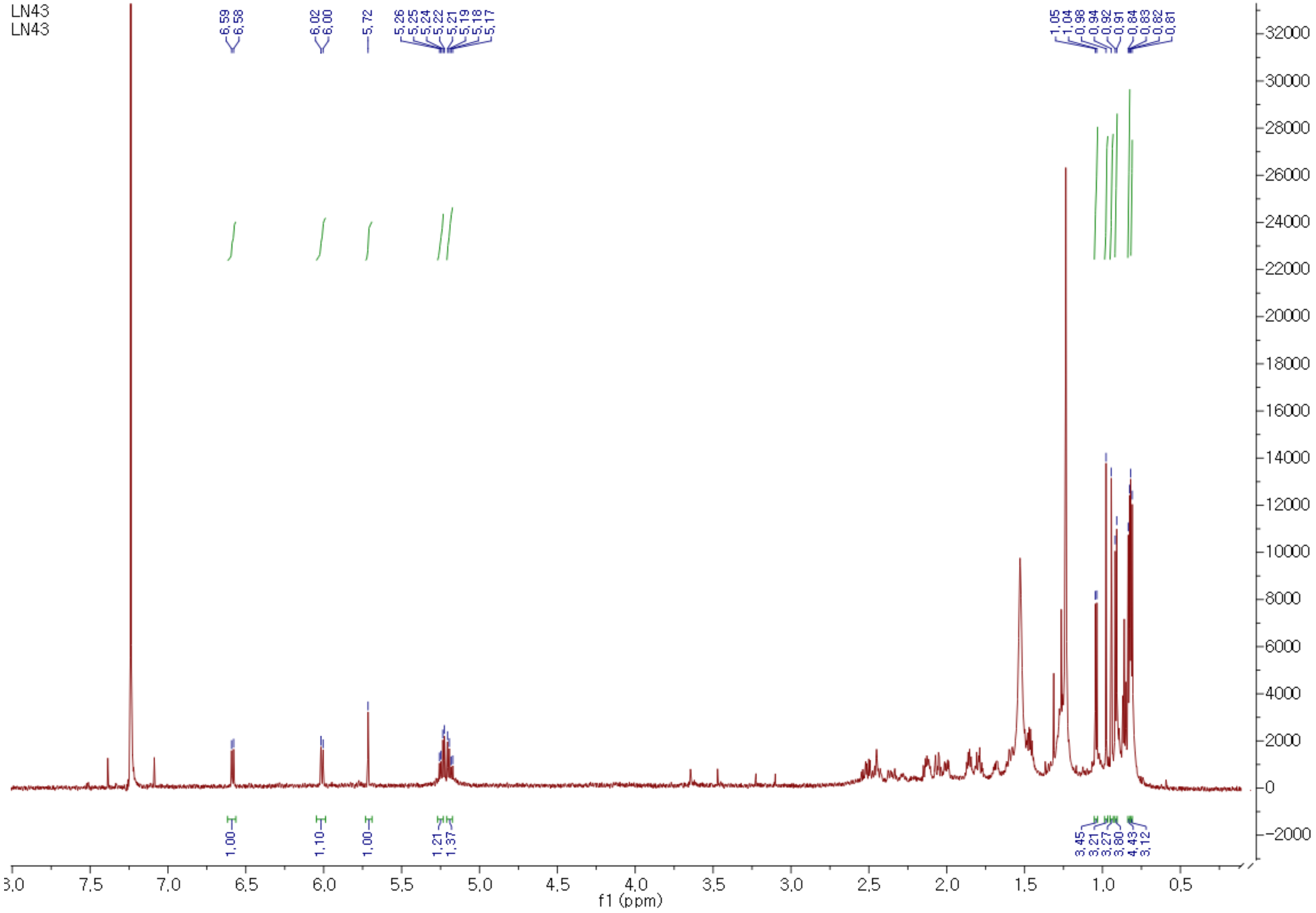

Figure S30. ^{1}H NMR spectrum of **14** ($CDCl_3$, 700 MHz)

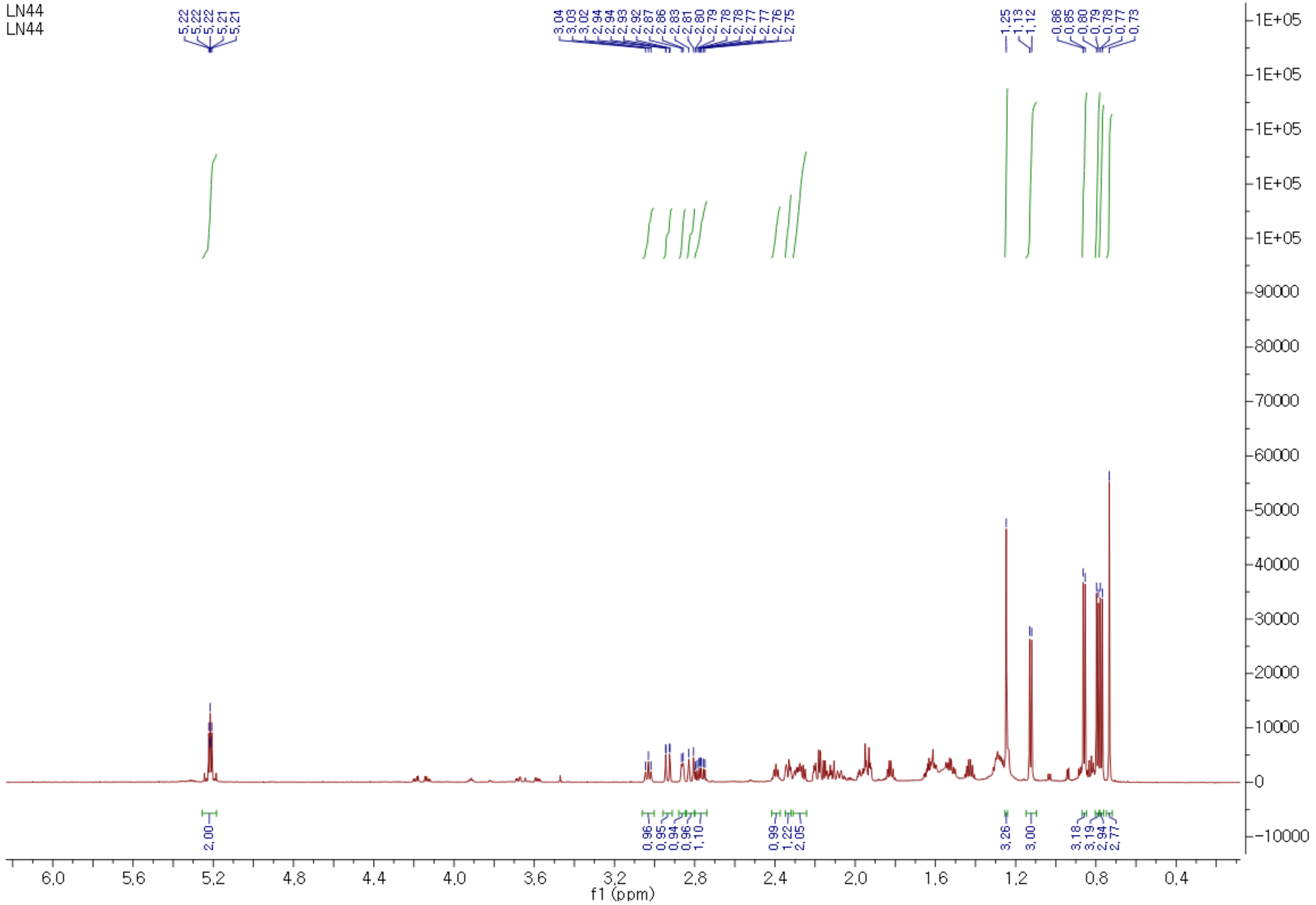

Figure S31. ^{13}C NMR spectrum of **14** ($CDCl_3$, 175 MHz)

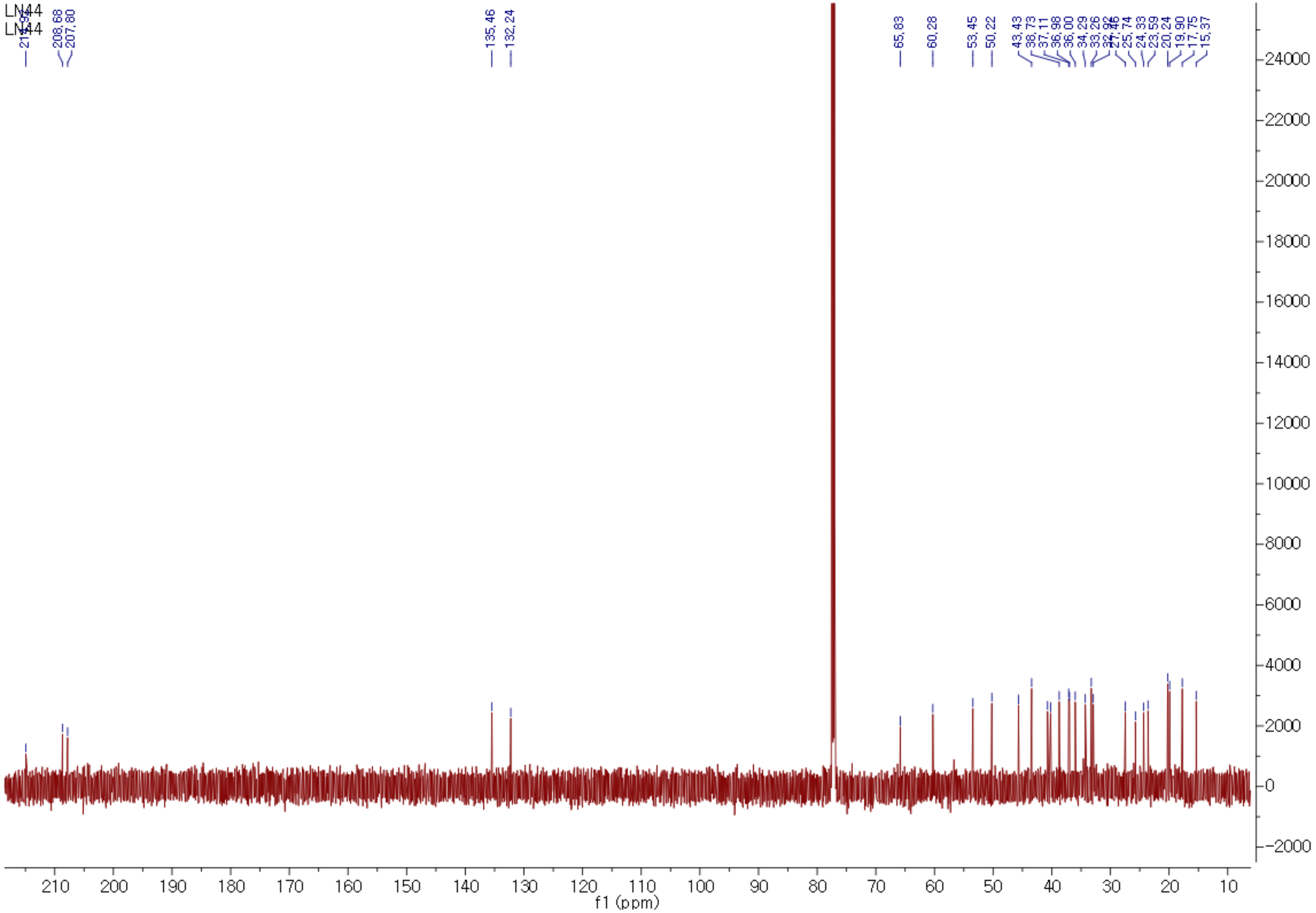

***In Vitro* Cytotoxicity Test.** A sulforhodamine B (SRB) bioassay was used to determine the cytotoxicity of compound **1** against human breast cancer cell lines (Bt549, HCC70, and MDA-MB-468). Each cell line was inoculated into standard 96-well, flat-bottom microplates and incubated for 24 h at 37 °C in a humidified atmosphere containing 5% CO_2. The attached cells were then incubated with serially diluted isolates. After continuous exposure to the compounds for 72 h, the culture medium was removed from each well and the cells were fixed with 10% cold trichloroacetic acid at 4 °C for 1 h. After washing with tap water, the cells were stained with 0.4% SRB dye and incubated for 30 min at room temperature. The cells were washed with a 1% acetic acid solution, solubilized with 10 mM unbuffered Tris base solution (pH 10.5), and the absorbance was measured at 520 nm using a microtiter plate reader. The IC_{50} of cancer cell growth was expressed as the mean from three independent experiments.
